# [^68^Ga]Ga-NODAGA-E[(cRGDyK)]_2_ angiogenesis PET following myocardial infarction in an experimental rat model predicts cardiac functional parameters and development of heart failure

**DOI:** 10.1007/s12350-023-03265-9

**Published:** 2023-05-01

**Authors:** Simon Bentsen, Jacob Kildevang Jensen, Esben Christensen, Lars Ringgaard Petersen, Constance Eline Grandjean, Bjarke Follin, Johanne Straarup Madsen, Camilla Christensen, Andreas Clemmensen, Tina Binderup, Philip Hasbak, Rasmus Sejersten Ripa, Andreas Kjaer

**Affiliations:** 1 Department of Clinical Physiology, Nuclear Medicine & PET and Cluster for Molecular Imaging, Department of Biomedical Sciences, Rigshospitalet and University of Copenhagen, Copenhagen, Denmark; 2 Department of Health Technology, Section for Biotherapeutic Engineering and Drug Targeting, Technical University of Denmark, Copenhagen, Denmark; 3 Cardiology Stem Cell Centre, The Heart Centre, Rigshospitalet, University of Copenhagen, Copenhagen, Denmark

**Keywords:** Myocardial biology, coronary artery disease, myocardial ischemia and infarction, positron emission tomography

## Abstract

**Background:**

Angiogenesis has increasingly been a target for imaging and treatment over the last decade. The integrin α_v_β_3_ is highly expressed in cells during angiogenesis and are therefore a promising target for imaging. In this study, we aimed to investigate the PET tracer [^68^Ga]Ga-RGD as a marker of angiogenesis following MI and its ability to predict cardiac functional parameters.

**Methods:**

First, the real-time interaction between [^68^Ga]Ga-RGD and integrin α_v_β_3_ was investigated using surface plasmon resonance (SPR). Second, an animal study was performed to investigate the [^68^Ga]Ga-RGD uptake in the infarcted area after one and four weeks following MI in a rat model (MI = 68, sham surgery = 36). Finally, the specificity of the [^68^Ga]Ga-RGD tracer was evaluated ex vivo using histology, autoradiography, gamma counting and flow cytometry.

**Results:**

SPR showed that [^68^Ga]Ga-RGD has a high affinity for integrin α_v_β_3_, forming a strong and stable binding. PET/CT showed a significantly higher uptake of [^68^Ga]Ga-RGD in the infarcted area compared to sham one week (*p* < 0.001) and four weeks (*p* < 0.001) after MI. The uptake of [^68^Ga]Ga-RGD after one week correlated to end diastolic volume (*r* = 0.74, *p* < 0.001) and ejection fraction (*r* = − 0.71, *p* < 0.001) after four weeks.

**Conclusion:**

This study demonstrates that [^68^Ga]Ga-RGD has a high affinity for integrin α_v_β_3_, which enables the evaluation of angiogenesis and remodeling. The [^68^Ga]Ga-RGD uptake after one week indicates that [^68^Ga]Ga-RGD may be used as an early predictor of cardiac functional parameters and possible development of heart failure after MI. These encouraging data supports the clinical translation and future use in MI patients.

**Supplementary Information:**

The online version contains supplementary material available at 10.1007/s12350-023-03265-9.

## Introduction

Adverse cardiac remodeling after myocardial infarction (MI) is a process of structural and functional changes which could lead to heart failure. Remodeling of the myocardium occurs as a response to inadequate repair after an MI.^1–3^ The repair of the myocardium after MI consists of three phases: the acute, the proliferation and scar maturation phase. During the proliferation phase, endothelial cells proliferate and infiltrate the infarcted area, leading to the formation of a dense microvascular network, which supplies oxygen and nutrients to the infarcted area.^4,5^ This process is known as angiogenesis and is essential to repair after MI.^6^ The third phase is the maturation phase, in which most of the myofibroblasts transition to a phenotype that promotes scar maturation.^3,7^ Angiogenesis peaks seven days after MI and slowly decreases over the next 14 to 28 days.^8^ Since angiogenesis is vital to repair, the targeting and stimulation of angiogenesis has been of clinical interest for many years.

To develop and optimize treatment that promotes angiogenesis, it is crucial to establish a non-invasive method for the real-time monitoring of angiogenesis. Positron emission tomography (PET) is a modality with high sensitivity and acceptable resolution that enables continuous in vivo monitoring of angiogenesis in human subjects. The integrin α_v_β_3_ has been extensively studied and the highest expression of this integrin has been found in activated endothelial cells undergoing angiogenesis.^9–11^ The integrin α_v_β_3_ is expressed at low levels in normal healthy tissue like intestinal, vascular and smooth muscle cells.^12^ Other cell types with expression of integrin α_v_β_3_ is bone resorbing osteoclasts, activated macrophages, angiogenic endothelial cells and migrating smooth muscle cells.^13^ The tripeptide motif Arg-Gly-Asp (RGD) is a specific ligand to α_v_β_3_ and has been the major peptide used in the molecular imaging of α_v_β_3_, since it is recognized by the α-subunit of the integrin^14,15^ on the endothelial cell. Several different RGD-based PET tracers have previously been used for imaging integrin, primarily in animal studies. The tracers differ in characteristics like linkers, chelators, radionuclides.^16–18^.

The aim of this study was to investigate the emerging PET radiotracer [^68^Ga]Ga-NODAGA-E[(cRGDyK)]_2_ ([^68^Ga]Ga-RGD), as a marker of angiogenesis and its potential use in predicting outcome following MI. This was done by *(a)* evaluating the binding between α_v_β_3_ and [^68^Ga]Ga-RGD using surface plasmon resonance (SPR), *(b) *in vivo animal experiment imaging angiogenesis in a myocardial infarction rodent model using [^68^Ga]Ga-RGD PET/CT and *(c) *ex vivo evaluation to confirm infarction (histology), cell distribution (flow cytometry) and verify tracer accumulation using autoradiography and gamma counting.

## Methods

### Study design

#### Binding kinetics

The assessment of real-time biomolecular interaction was performed using a Biacore X100 (Biacore, Uppsala, Sweden), with the determination of binding kinetics of [^68^Ga]Ga-RGD and integrin α_v_β_3_. To evaluate the assay development and results, vitronectin and fibronectin was used as comparison.

#### Ethical statement

The Danish Animal Experiments Inspectorate approved experimental protocols (Permit No. 2016-15-0201-00920). All animal procedures performed are in accordance with the guidelines in Directive 2010/63/EU of the European Parliament on the protection of animals used for scientific purposes.

#### Animal experiment

**Figure 1:**
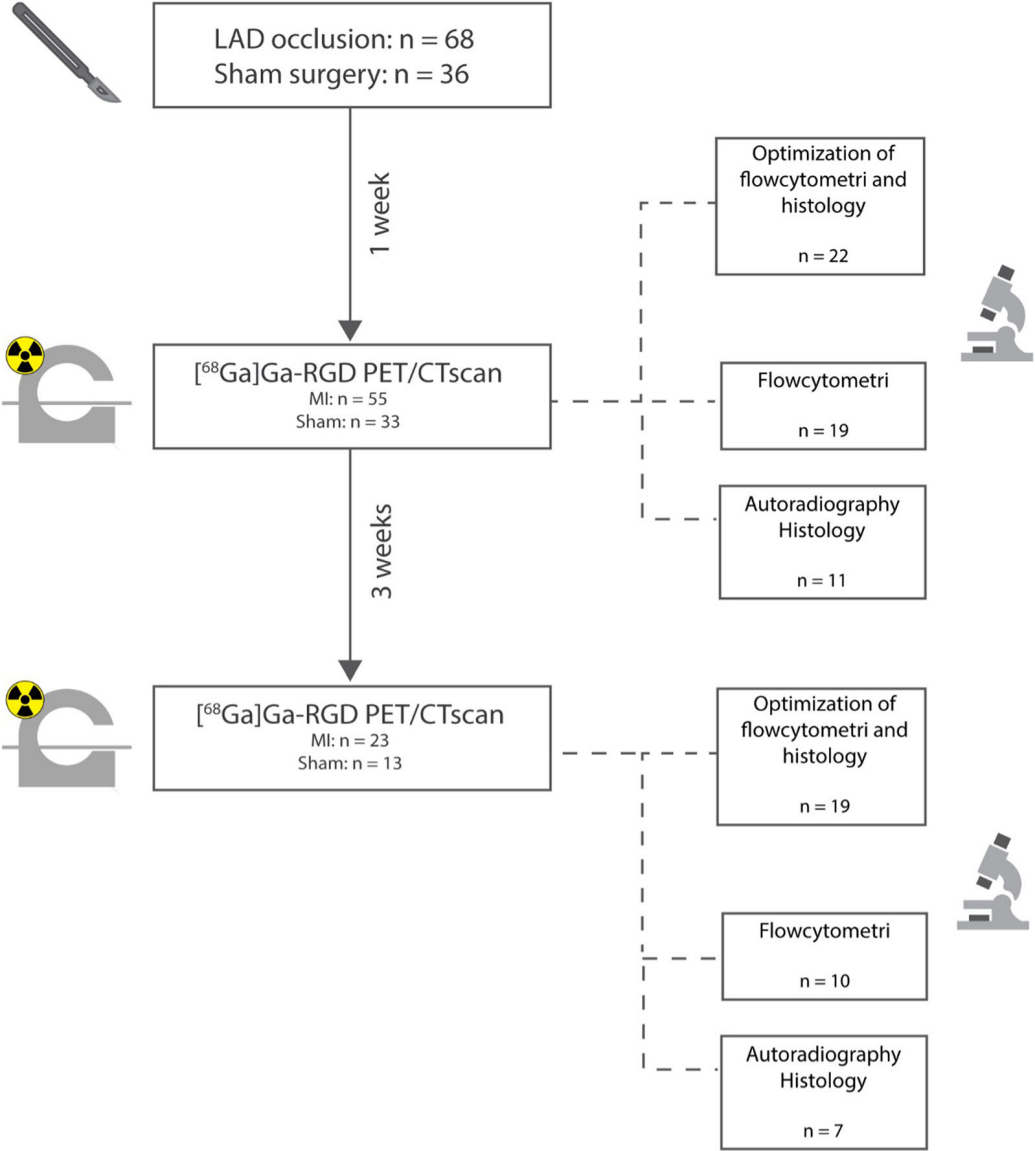
Workflow of the study. At first, the rats underwent either sham or permanent LAD-occlusion. The rats were PET/CT scanned after 1 week and follow-up scanned 4 weeks after operation. *LAD*, left anterior descending coronary artery; *[*^*68*^*Ga]Ga-RGD*, [^68^Ga]Ga-NODAGA-E[(cRGDyK)]_2_; *PET*, positron emission tomography; *CT*, computed tomography

104 animals were included and underwent open chest surgery. The rats were randomly assigned to either sham or LAD ligation to induce an experimental myocardial infarction in a 1:3 ratio. One week after operation, the surviving rats (n = 88) were PET/CT scanned with 2-deoxy-2-[^18^F]fluoro-D-glucose (2-[^18^F]FDG) and the following day [^68^Ga]Ga-RGD. After the [^68^Ga]Ga-RGD scan 52 of the rats were euthanized and the excised heart used for flow cytometry or autoradiography and histology. Four weeks after the operation, a follow-up scan of 2-[^18^F]FDG and [^68^Ga]Ga-RGD were performed (n = 36). Following [^68^Ga]Ga-RGD PET/CT scan the animals were euthanized, and myocardial tissue used for either flow cytometry or histology and autoradiography (flowchart depicted in Figure 1).

See supplementary for full description of methods.

## Results

### Integrin α_v_β_3_, SPR experiments by single cycle kinetics

**Figure 2:**
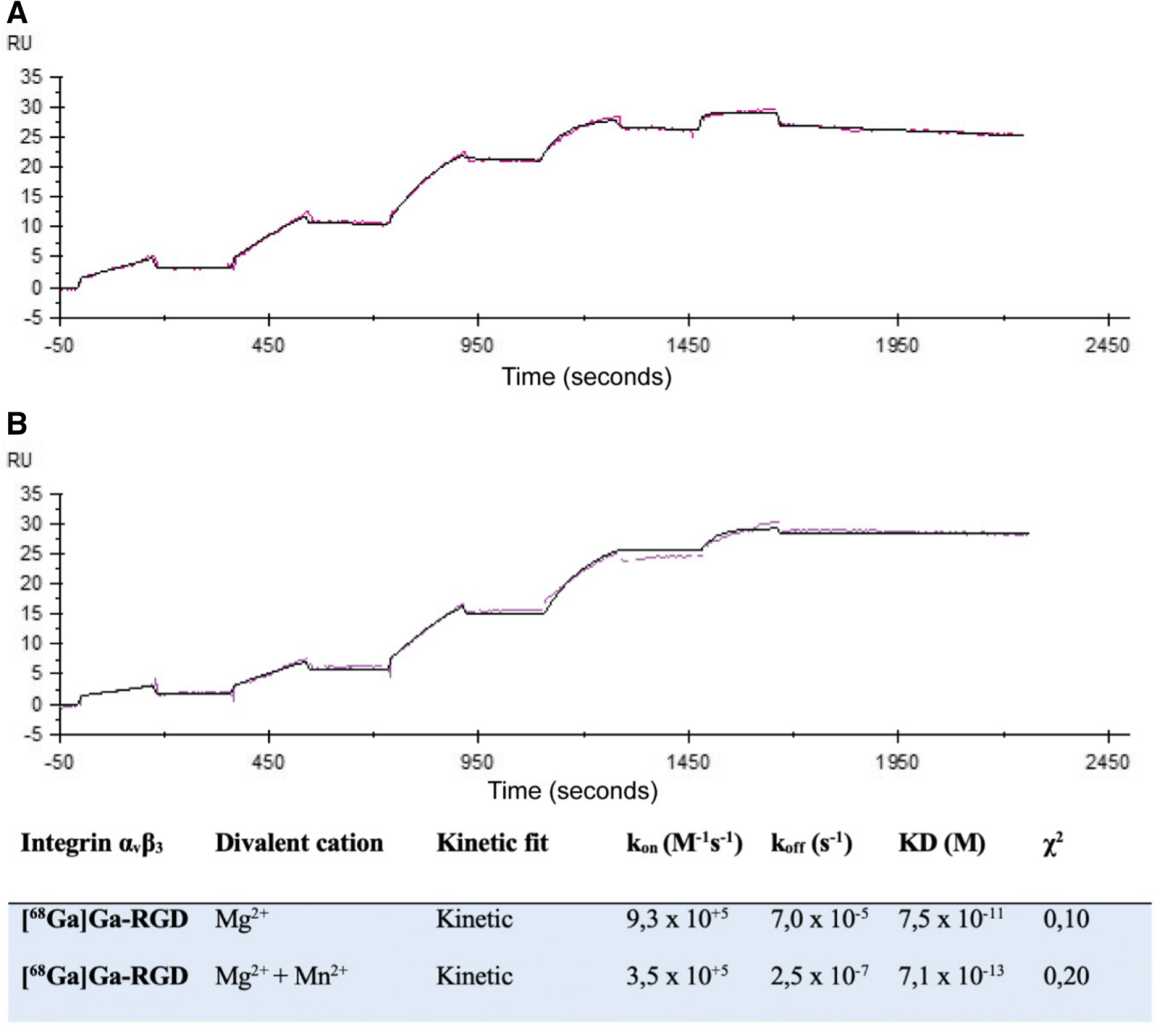
Sensorgrams of the interaction between [^68^Ga]Ga-RGD and integrin α_v_β_3_ in the present of Mg^2+^ (**A**) and Mg^2+^ and Mn^2+^ (**B**). The measured response is shown as the red curve, while the fitted model is the black. *RU*, response unit

The binding between integrin α_v_β_3_ and [^68^Ga]Ga-RGD showed a strong and stable interaction in the presence of Mg^2+^ and an even slower dissociation rate constant in the presence of Mg^2+^ and Mn^2+^ (Figure 2).

Integrin α_v_β_3_ is the natural receptor for vitronectin, and SPR showed likewise a strong interaction with vitronectin in the presence of Mg^2+^ with no change in the presence of Mg^2+^ and Mn^2+^ and thereby validate the SPR analyses of integrin α_v_β_3_ and [^68^Ga]Ga-RGD.

Fibronectin showed steady state interaction with integrin α_v_β_3_. (supplementary figure S1 and table S1).

The glycoprotein CD4 + was used as a control ligand. This would demonstrate if [^68^Ga]Ga-RGD binds unspecific in the present of a protein on the CM5 chip. There was no interaction between CD4 + and [^68^Ga]Ga-RGD, indicating that [^68^Ga]Ga-RGD does not have unspecific binding in the presence of a protein.

### Animal experiments

#### Myocardial infarction extent

In 1 examination out of the total 124 (0.8%), 2-[^18^F]FDG-PET were of poor quality and excluded from the dataset(1 week: sham = 1). In the MI group, PET showed a lack of 2-[^18^F]FDG uptake in the anterolateral wall of the left ventricle corresponding to the infarcted area. As expected, the extent of the defect was higher in the MI group, compared to sham after one week (*p* < 0.001). After four weeks, the extent of the defect in the MI group was still larger than sham (*p* < 0.001). (Table 1).

**Table 1:** Baseline and follow-up characteristic in PET/CT

	1 week			4 weeks		
	Infarct	Sham	P	Infarct	Sham	P
	N = 55	N = 33		N = 23	N = 12	
2-[^18^F]FDG-PET						
Infarct extent (%)	14.5 ± 1.2	1.6 ± 0.3	< 0.001	18 ± 2	2.5 ± 0.5	< 0.001
Left ventricular end diastolic volume, (µl)	476 ± 10	437 ± 10	0.015	608 ± 20	491 ± 16	< 0.001
Left ventricular end-systolic volume, (µl)	215 ± 9	148 ± 5	< 0.001	256 ± 13	161 ± 11	< 0.001
Left ventricular ejection fraction, %	54 ± 1.4	66 ± 0.8	< 0.001	58 ± 1	67 ± 1.3	< 0.001
[^68^Ga]Ga-RGD PET						
%ID/g, anterior wall	0.15 ± 0.005	0.09 ± 0.003	< 0.001	0.11 ± 0.005	0.06 ± 0.003	< 0.001
%ID/g, posterior wall	0.09 ± 0.003	0.09 ± 0.003	0.4	0.07 ± 0.002	0.07 ± 0.002	0.2
%ID/g, blood pool	0.09 ± 0.005	0.07 ± 0.006	0.1	0.07 ± 0.008	0.06 ± 0.004	0.7
Target-to-background ratio	1.6 ± 0.3	0.9 ± 0.02	< 0.001	1.5 ± 0.05	0.8 ± 0.03	< 0.001

*%ID/g*, % injected dose per gram tissue

#### In vivo [^68^Ga]Ga-RGD PET-uptake

PET-uptake measurements of [^68^Ga]Ga-RGD in %ID/g are shown in Table 1. The intraclass correlation coefficient for %ID/g of the anterior wall (0.84; 95% CI 0.75–0.9) and posterior wall (0.86; CI 95% 0.79–0,91) measurements were good.

**Figure 3:**
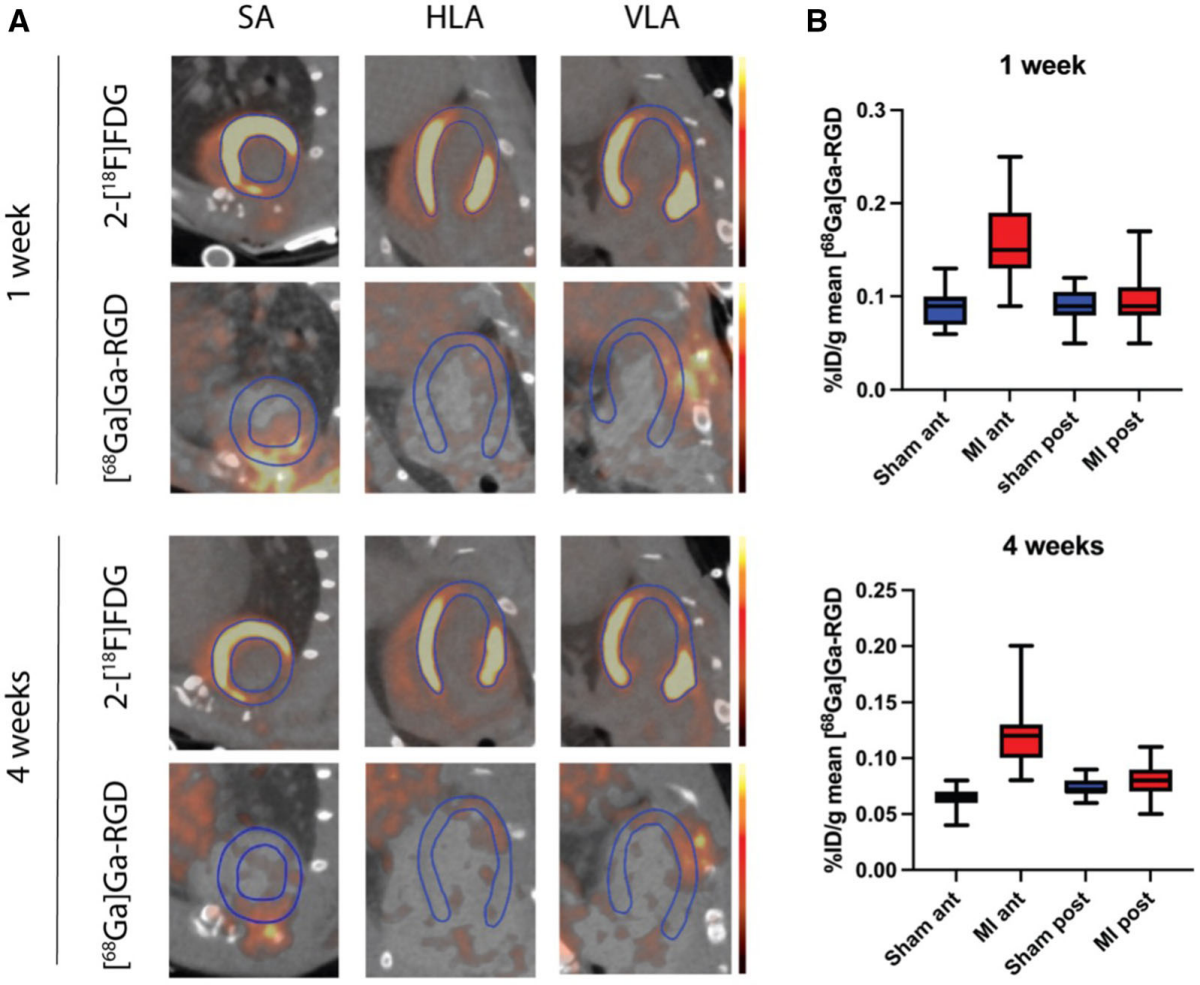
Representative PET/CT images of the same rat one and four weeks after surgery. The 2-[^18^F]FDG -PET shows a significant lack of tracer uptake in the anterolateral parts of the myocardium at one week and four weeks post-surgery. The [^68^Ga]Ga-RGD PET shows that the uptake corresponds to the area with a lack of tracer uptake on the 2-[^18^F]FDG-PET. *SA*, short axis view; *VLA*, vertical axis view; *HLA*, horizontal axis view. Other abbreviations as in previous figures

One week after total chronic occlusion of the LAD, the MI group showed a significantly higher uptake of [^68^Ga]Ga-RGD by in vivo PET, compared to the sham group in the anterior wall of the left ventricle (LV) (Supplementary figure S4). After four weeks, the MI group still showed a significantly higher uptake of the [^68^Ga]Ga-RGD in the anterior wall, compared to sham animals. In the non-infarcted posterior LV wall, there was no difference in uptake of [^68^Ga]Ga-RGD after one and four weeks, when comparing the MI group with the sham group (Figure 3).

#### EDV and EF measured from gated 2-[^18^F]FDG-PET and correlation to [^68^Ga]Ga-RGD

**Figure 4:**
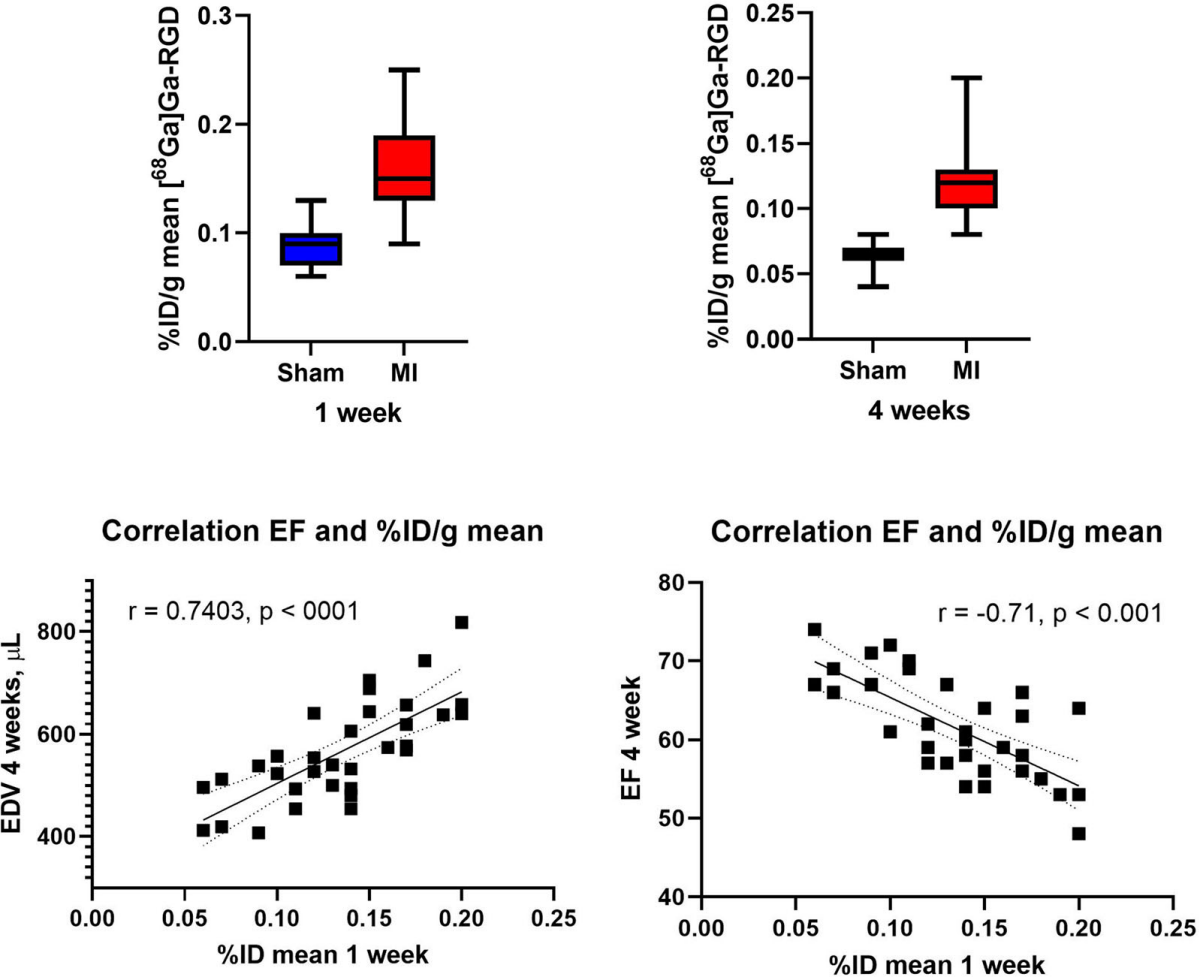
[^68^Ga]Ga-RGD uptake in the anterior wall of the LV after one week (**A**) and four weeks (**B**). The uptake is higher after one week, but there is no difference between the MI groups between week one and week four. There is a positive correlation between EDV at four weeks and uptake of [^68^Ga]Ga-RGD after one week (**C**). There is a negative correlation between EF at four weeks and uptake of [^68^Ga]Ga-RGD after one week (**D**). *EDV*, end diastolic volume; *EF*, ejection fraction; *FU*, follow-up. Other abbreviations as in previous figures

ECG-gated 2-[^18^F]FDG-PET/CT was used to analyze EDV, ESV and EF. The MI groups had a significantly higher EDV after one week compared to the sham group (*p* = 0.015). After four weeks the EDV in the MI group had increases and was significantly higher than the sham, indicating a progression towards a heart failure phenotype in the MI group (Table 1). The MI group had a significantly lower EF, compared to sham after one week. After four weeks, the difference in EF was still evident. There was no difference in EF from one week to four-week scan between the groups (Table 1). There was a positive correlation between mean [^68^Ga]Ga-RGD uptake (%ID/g) in the anterior LV wall after one week and the four weeks end diastolic volume (*r* = 0.74, *p* < 0.001). A negative correlation was observed between max [^68^Ga]Ga-RGD uptake (%ID/g) at one week and EF at four weeks (*r* = -0.71, *p* < 0.001) (Figure 4).

In multiple regression analysis with EDV after four weeks, only %ID/g is significant in predicting EDV after four weeks (%ID/g *p* = 0.005, LVEF acute *p* = 0.305, FDG extent acute *p* = 0.127).

#### Uptake of [^68^Ga]Ga-RGD following myocardial infarction correlates with α_v_ 1 expression on endothelial cells.

**Figure 5:**
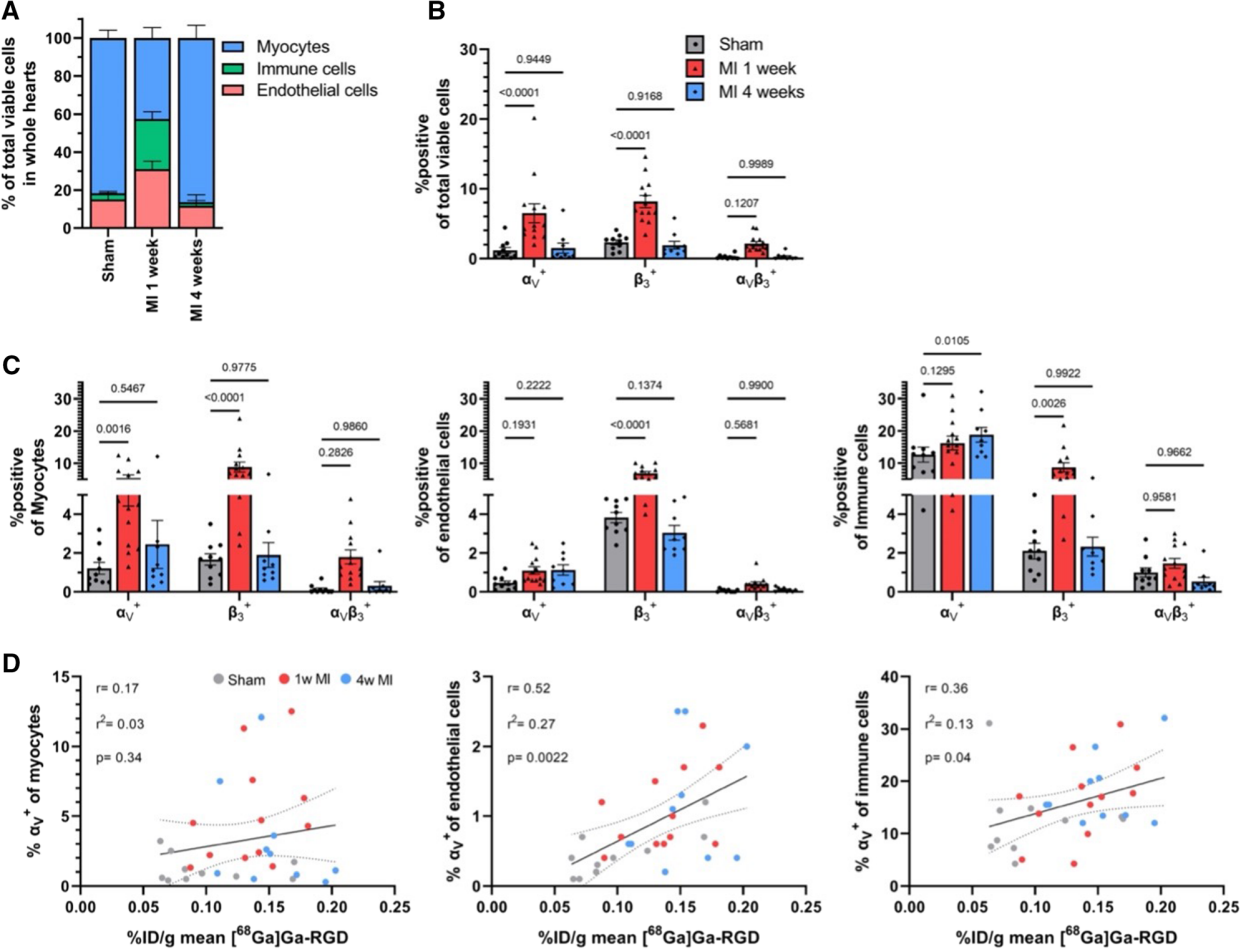
Flow cytometry evaluation of heart after MI. Cellular composition of hearts (**A**). Positivity of α_v_ and β_3_ of all cells in hearts (**B**). α_v_ and β_3_ expression on endothelial cells (**C**), myocytes, and immune cells. Correlation of [^68^Ga]Ga-RGD uptake to α_v_ on endothelial cells, myocytes and immune cells (**D**). n = 12 one week after MI, n = 12 four weeks after MI and n = 10 for sham. Statistical differences were determined using mixed-effect analyses with Dunnetts correction for multiple comparisons to sham in **A**–**C** and pearsons correlation in **D**. *MI*, myocardial infarction; *%ID/g*, % injected dose per gram tissue. Other abbreviations as in previous figures

Cellular distribution of α_v_β_3_ was evaluated in whole rat hearts by flow cytometry one and four weeks after MI. One week after MI there was an increase in the amount of immune cells and endothelial cells compared to the sham group (*p* = 0.0006 and 0.018, respectively) which four weeks after MI returned to the cellular composition to the levels of sham treated animals (*p* > 0.05 for all cell types; Figure 5A). The overall amount of cells positive for α_v_β_3_ across cell types increased drastically 1 week after MI (*p* < 0.0001) and returned to sham levels 4 weeks after MI (*p* > 0.05; Figure 5B). Besides the changes in cellular composition of the heart and the overall increase in α_v_ and β_3_ one week after MI, myocytes increased their expression of α_v_ and β_3_ (*p* = 0.0016 and < 0.0001, respectively) while only β_3_ was increased on endothelial cells and immune cells (*p* < 0.0001 and = 0.0026, respectively). The increased expression of β_3_ returned to sham levels in all cell types four weeks after MI (*p* > 0.05) while α_v_ had a statistically insignificant trend towards being slightly increased across cell types (Figure 5C).

Correlating the investigated cellular findings to the mean %ID/g [^68^Ga]Ga-RGD uptake revealed the best correlation for α_v_ expression on endothelial cells (*r* = 0.52, *P* = 0.002) which was less apparent on immune cells and myocytes (Figure 5D) as well as other possible correlations (Supplementary Figure S2).

#### Histological analysis of the heart

**Figure 6:**
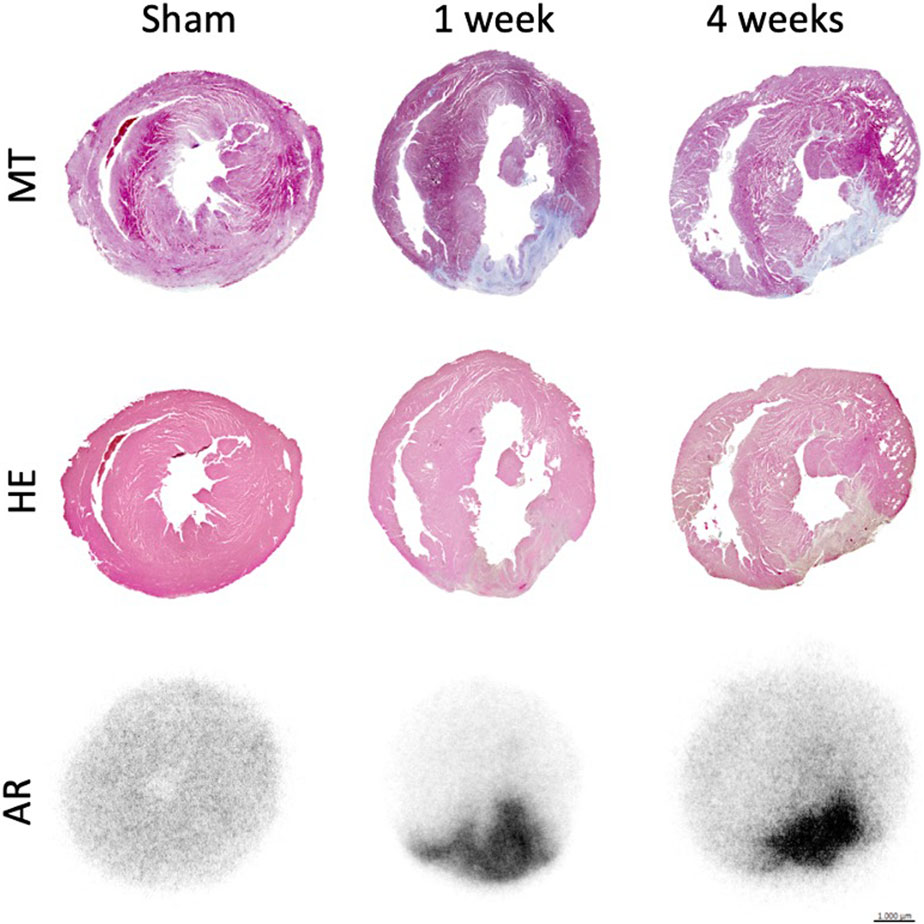
Histological verification of infarction after one and four weeks. The staining for Masson’s trichrome shows a fibrotic area corresponding to the accumulation of [^68^Ga]Ga-RGD pictured with autoradiography. *MT*, Masson’s Trichrome; *HE*, Hematoxylin & Eosin; *AR*, autoradiography

**Figure 7:**
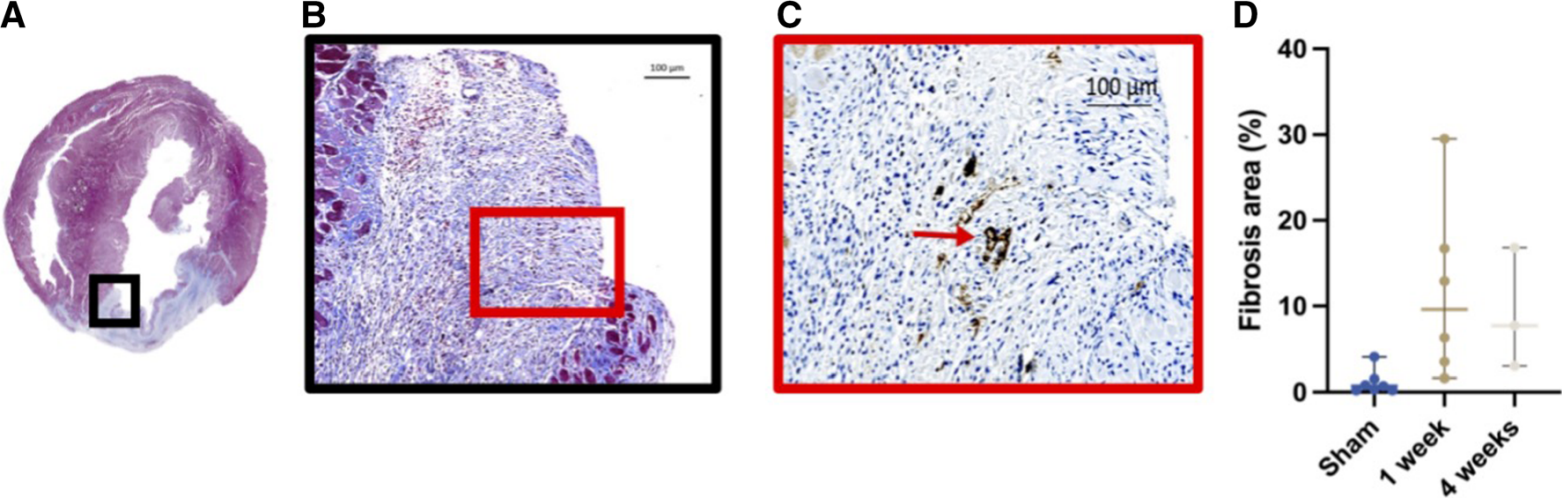
Masson’s trichrome staining shows a clear area of fibrosis compared to healthy tissue (**A**, **B**). The immunohistochemically staining for integrin β_3_ shows an accumulation in the vessel wall (**C**). The quantification of fibrotic tissue on MT shows a significant difference between sham and the MI groups (**D**). The extent of fibrosis is unchanged between week 1 and 4 in the MI group, indicating a stable and consistent ligation of the LAD

The presence of an infarcted area in the myocardium was confirmed by histological evaluation of the heart. The infarcted area on HE stains showed regions with a lack of myocytes and many cells with atypical nuclei (Figure 6). This was more profound and transmural at four weeks compared to after one week. MT stains showed collagen accumulation (fibrosis) in the infarcted area, represented as blue staining. This was more delineated at four weeks compared to after one week. When measuring fibrosis density (percentage of total area) on MT-stained cross-sections of the heart (Figure 7 A,B), there was a significantly higher degree of fibrosis in the MI group, compared to sham after one week and after four weeks (sham: 1.1 ± 1.3; one week: 11.7 ± 10; four weeks: 9.1 ± 7.0, *p* = 0.047, Figure 7 D).

Immunohistochemically staining for the integrin β_3_ chain showed a stronger positivity for β_3_ in infarcted tissue compared to healthy tissue (Figure 7 C, red arrow), with the β_3_ being primarily present in the wall of the vessel.

#### [^68^Ga]Ga-RGD uptake after ischemia measured by autoradiography and gamma counting of the heart ex vivo

For autoradiography analysis, the sham group, showed no focal [^68^Ga]Ga-RGD accumulation in the myocardium, only homogeneous background activity in the myocardium. In the MI group, an increased focal [^68^Ga]Ga-RGD uptake was observed, corresponding to the infarcted area. This was evident both one week and four weeks after MI. Gamma counting showed a significantly higher %ID/g of [^68^Ga]Ga-RGD in the MI group, compared to the sham group. After one week, the mean %ID/g were 0.12 vs 0.068 (*p* < 0.001) and after four weeks 0.11 vs 0.063 %ID/g (*p* < 0.001). There was a significant correlation between gamma counting and autoradiography (*r* = 0.62, *p* < 0.001).

## Discussion

The major finding of this study is that [^68^Ga]Ga-RGD PET-uptake in the infarcted area correlates with cardiac functional parameters at a later time point. More specifically, we found that the uptake of [^68^Ga]Ga-RGD in the infarcted area after one week correlated to the EDV and EF measured after four weeks. The [^68^Ga]Ga-RGD tracer used was recently developed by our group,^19–22^ and demonstrated in the present study, a stronger binding to α_v_β_3_ than that of its natural ligand vitronectin. We also demonstrated that after a MI, endothelial cells express a higher level of α_v_β_3_ after one week, declining after four weeks. The focal uptake of [^68^Ga]Ga-RGD on autoradiography was present in the infarcted area and not in healthy myocardium_,_ correlating to measured [^68^Ga]Ga-RGD counts in the respective slices of the heart. In vivo imaging of angiogenesis using [^68^Ga]Ga-RGD with PET/CT showed a higher uptake of [^68^Ga]Ga-RGD in the infarcted area compared to sham after both one and four weeks.

Our study is not the first to image angiogenesis based on integrin α_v_β_3_ targeted PET tracer in a rat model of acute MI. However, in this study, we present in vivo and ex vivo data that support that binding between integrin α_v_β_3_ and [^68^Ga]Ga-RGD is better than previously for other RGD targeting PET tracers, *e.g.,* ^64^Cu-NOTA-PEG_4_-cRGD, a cyclic RGD-peptide radiolabelled with ^64^Cu for PET imaging.^16^ This radiolabelled version has a different chelator (NOTA-PEG_4_), a different isotope (^64^Cu) and used a cyclic RGD-peptide rather than the dimeric RGD-peptide radiolabelled version used in our study (chelator: NODAGA, isotope: ^68^Ga). We previously showed that the dimeric [^68^Ga]Ga-NODAGA-E[(cRGDyK)]_2_ has a higher affinity towards integrin α_v_β_3_ than the monomer.^23^ SPR analysis of ^64^Cu-NOTA-PEG_4_-cRGD and integrin α_v_β_3_ showed the same KD as we present,^16^ but the dissociation rate is slower between our [^68^Ga]Ga-RGD and integrin α_v_β_3_ than ^64^Cu-NOTA-PEG_4_-cRGD and integrin α_v_β_3_. This could indicate a more stable complex between [^68^Ga]Ga-RGD and integrin α_v_β_3_ than ^64^Cu-NOTA-PEG_4_-cRGD and integrin α_v_β_3_.

SPR has previously been used to assess the binding between integrin α_v_β_3_ and RGD.^24^ This study found that the binding between integrin α_v_β_3_ and vitronectin was improved with the presence of Mn^2+^. Our SPR analysis of vitronectin and integrin α_v_β_3_ showed the same level of affinity, which confirms the accuracy of our setup. We found that binding of [^68^Ga]Ga-RGD to integrin α_v_β_3_ was 1000-fold stronger than vitronectin in the presence of Mn^2+^, suggesting that [^68^Ga]Ga-RGD binds specifically and with a stable complex to integrin α_v_β_3_.

Flow cytometry analysis of the heart showed that the level of α_v_β_3_ positive cells were significantly higher after one week in the MI group compared to the sham in the myocytes/myofibroblasts, immune cells, and endothelial cells. The literature describes α_v_β_3_ to be involved with different actions of the different cell types, all which relates to myocardial wound healing, which is activation and migration of endothelial cells, infiltration and anti-inflammatory actions for immune cells,^25^ resistance to apoptosis for myocytes^26^ and matrix remodeling for myofibroblasts.^27^ The flow cytometry analysis supports the many different actions of α_v_β_3_, since the levels of positive α_v_β_3_ cells were present in myocytes, immune cells and endothelial cells. However, the best correlation was between RGD uptake and endothelial cells, which indicates that even though the integrin is present in other cell types relating to myocardial wound healing, the signal detected by RGD PET is mostly derived from angiogenesis.

The percentages of α_v_β_3_ positive immune cells and endothelial cells both declined from one to four weeks after MI, indicating that the immunomodulation and formation of new blood vessels had declined. The decline in α_v_β_3_ positive cells by flow cytometry was in our study paralleled by a decline in in vivo uptake of [^68^Ga]Ga-RGD.

In vivo PET imaging showed a significant increase in [^68^Ga]Ga-RGD uptake in the infarcted myocardium, compared to non-infarcted myocardium. This was evident both at one and four weeks after MI which correlated best with α_v_ expression on endothelial cells, indicating that [^68^Ga]Ga-RGD is a promising PET tracer in detecting angiogenesis and remodeling. The first scan was performed after one week, to ensure that the initial inflammatory response following MI did not affect the [^68^Ga]Ga-RGD uptake. The follow-up scan was done after four weeks, to investigate the potential shift from angiogenesis to remodeling. Several other studies have shown angiogenesis imaged with other RGD PET tracers^17,18,28–30^ and three clinical trial has been performed.^31–33^ However, there are some inconsistency towards the conclusions of these studies. Sherif et al. performed a preclinical trial with permanent ligation of the LAD artery and F-galatco-RGD 1 week after MI. In this study a low uptake of RGD was associated with lower EF and higher EDV. Jenkins et al. showed that in 21 patient with ST-elevation myocardial infarction, high uptake of F-fluciclatide RGD was predicting regions of recovery.

It is established that left ventricular dilation is correlated with subsequent heart failure independently of risk factors and EF in humans.^34^ Our findings of a correlation between EDV at four weeks follow-up with [^68^Ga]Ga-RGD uptake one week after MI, indicate that [^68^Ga]Ga-RGD could be used as a non-invasive method to early identify patients at risk of developing dilated cardiomyopathy following a MI. The clinical implications of such a tool may be paramount. While it seems documented in our study that [^68^Ga]Ga-RGD is increased in the presence of MI with high sensitivity and specificity, further studies are needed to confirm the correlation between early uptake of [^68^Ga]Ga-RGD and subsequent adverse remodeling.

One of the great advantages of the [^68^Ga]Ga-RGD tracer used in the present study is the use of a generator produced radionuclide, which circumvents the need of an on-site cyclotron. The production of [^68^Ga]Ga-NODAGA-E[(cRGDyK)]_2_ is fast, simple, with a high yield, and the PET tracer is stable. Other RGD PET tracers, such as those labeled with ^18^F, need a cyclotron close by.

## Study limitations

This study investigated permanent ligation of the LAD as a MI model. To assess the broader applicability of [^68^Ga]Ga-RGD studies in reperfusion models with transient occlusion of the LAD should be conducted in the future. A reperfusion model would be a more clinically relevant and translate better to investigate acute myocardial infarction in humans.

This study was not designed to investigate heart failure following MI. To establish if [^68^Ga]Ga-RGD uptake correlates with heart failure, the animals needed to be observed for a longer period of time.

In this study the functional cardiac parameters were EDV, ESV and EF. To assess the cardiac function in greater detail, magnetic resonance imaging or a perfusion tracer such as ^13^N-NH_3_ could be used to evaluate myocardial blood flow and myocardial flow reserve. However, ^13^N-NH_3_ requires an on-site cyclotron, making it difficult to examine in a rodent model.^35^

## Conclusion

This study demonstrates that [^68^Ga]Ga-RGD has a high affinity for integrin α_v_β_3_ which enables the evaluation of angiogenesis following an MI, using PET/CT. The in vivo RGD uptake after one week correlated to ejection fraction and end diastolic volume after four weeks, indicating that [^68^Ga]Ga-RGD may be used as an early predictor of cardiac functional parameters and possible development of heart failure after an MI. These encouraging data support a clinical translation.

## New knowledge gained:

The PET tracer [^68^Ga]Ga-NODAGA-E[(cRGDyK)]_2_ forms a stable and strong binding to the integrin α_v_β_3_ α_v_β_3,_ which enable the detection of angiogenesis. The angiogenic response after one week correlated to predictors of early onset of heart failure phenotype.

### Supplementary Information

Below is the link to the electronic supplementary material.

Supplementary file1 (DOCX 1836 kb)

Supplementary file2 (PPTX 7627 kb)

## Abbreviations

AHA: American Heart Association
EDV: End diastolic volume
EF: Ejection fraction
ESV: End systolic volume
HE: Hematoxylin & Eosin
KD: Dissociation constant (M)
k_off_: Dissociation rate constant (s^−^^1^)
k_on_: Association rate constant (M^−^^1^ s^−^^1^)
LAD: Left anterior descending coronary artery
MI: Myocardial infarction
MT: Masson’s Trichrome
PET: Positron emission tomography
ROI: Region of interest
SPR: Surface plasmon resonance
%ID/g: Percentage injected dose per gram
2-[^18^F]FDG: 2-Deoxy-2-[^18^F]fluoro-D-glucose
[^68^Ga]Ga-RGD: [^68^ Ga]Ga-NODAGA-E[(cRGDyK)]_2_
