# Supplementary material for: [68Ga]Ga-NODAGA-E[(cRGDyK)]2 angiogenesis PET following myocardial infarction in an experimental rat model predicts cardiac functional parameters and development of heart failure
Source: J Nucl Cardiol. 2023 May 1;30(5):2073–84. doi: 10.1007/s12350-023-03265-9 (PMC10558373; doi:10.1007/s12350-023-03265-9)

Supplementary:

*In vitro and ex vivo experiments:*

*Surface plasmon resonance (SPR) studies*

The binding kinetics of ^68^Ga-RGD and integrin α_v_β_3_ was investigated. To evaluate the assay development and results, vitronectin and fibronectin was used as comparison. Vitronectin is the natural ligand for α_v_β_3_.

The assessment of real-time biomolecular interaction was performed using a Biacore X100 (Biacore, Uppsala, Sweden) on a CM5 sensor chip (Cytiva, Brønshøj, Denmark). Immobilization of the integrin α_v_β_3_ (R&D Systems, Minneapolis, USA) was done using an amine coupling kit (Cytiva Brønshøj, Denmark). Immobilization was performed at 25ºC using a HEPES buffer (10 mM HEPES, 150 mM Nacl, 3 mM EDTA, 0.05% P20, pH 7.4) as a running buffer. Flow cell 1 was the reference flow cell. Flow cell 1 was activated with 1-Ethyl-3-(3-dimethylaminopropyl) carbodiimide hydrochloride (EDC) and N-Hydroxysuccinimide (NHS) and deactivated with ethanolamine. Flow cell 2 was the active flow cell. Flow cell 2 was activated using EDC/NHC and integrins (20µg/mL) were flowed through the flow cell to react with the surface at a flow rate of 5µL/min, to achieve the calculated immobilization level (α_v_β_3_ ~ 4200 resonance unit (RU)). Immobilization of α_v_β_3_ was performed in 0.1 M sodium acetate pH 4.0. After immobilization three different analytes were examined: [^68^Ga]Ga-RGD, vitronectin (R&D systems, Minneapolis, USA) and fibronectin (R&D systems, Minneapolis, USA). Integrin and analyte was examined in two different running buffers. The first buffer included Tris-HCL 20mM, Nacl 150 mM, P20 0.05% and MgCl_2_ 1 mM pH 7.4 and the second buffer included Tris-HCL 20mM, Nacl 150 mM, P20 0.05%, MgCl_2_ 1 mM and MnCl_2_ 1 mM pH 7.4. The pH was adjusted with 0.1 M NaOH slowly, to inhibit MnCl_2_ to precipitate. First a series of multi-cycle kinetics experiments were done to assess the concentration range for the experiments. This showed that the dissociation was extremely slow and regeneration needed to run multiple experiments on the same chip. Various regeneration buffer were investigated (glycine-HCL 1.5 to 3 pH, 0-100% ethylene glycol, 1-100mM NaOH, 1-4 M MgCl_2_, 0.5-5 M NaCl_2_). None of the regeneration buffers could successfully remove the analyte without damaging the sensor chip. Therefore, all experiments was done on a freshly immobilized chip with single cycle kinetics. At least three independent experiments were performed per analyte.

To investigate if the interaction of [^68^Ga]Ga-RGD was specific to α_v_β_3_ or to the surface, glycoprotein CD4+ was immobilized and examined with single cycle kinetics.

*SPR analysis and evaluation*

SPR sensorgrams were analysed using Biacore X100 Evaluation Software 2.0.2 (Uppsala, Sweden). Reference cell responses was subtracted from corresponding active response curves. The resulting curves were then analysed and fit to the appropriate kinetic models. Globally fitted parameters were determined for each kinetic data set, with determination of association (k_on_), dissociation (k_off_) and dissociation constant (KD). Goodness of fit for each model was evaluated by analysing the residual plots and residual sum of squares (χ^2^). If the interaction displayed steady state interaction, only KD was determined. All data are shown in supplementary table s1

*Flow cytometry following MI*

Flow cytometry of rat hearts were performed one week and four weeks after surgery. Flow cytometric evaluation of the excised heart was done as previously described from our group ^18^. Briefly, the excised heart was perfused with isotonic saline immediately after extraction and placed in tissue storage. The tissue was transferred to tubes containing enzyme mix and subsequently filtered. An 8-color flow cytometry-based optimized multicolor immunofluorescence panel was used to evaluate the angiogenic response (supplementary table s2). The samples were acquired on LSR Fortessa X-20 (BD Biosciences, Kongens Lyngby, Denmark) and analyzed in FlowJo (BD Biosciences, Kongens Lyngby, Denmark). All populations were based on time, singlet, scatter and viability gating (figure S3). Endothelial cells were further defined as CD45^-^ CD31^+^. Myocytes were further defined as CD45^-^ CD31^-^. Immune cells were further defined as CD45^+^.

*Autoradiography and gamma counting of ^68^Ga-RGD*

Autoradiography and gamma counting of the rat hearts were performed one week (N: MI=6, sham=5) and four weeks (N: MI=4, sham=3) after surgery. The heart was excised and divided into four slices from apex to base. A dilution series were placed next to the slices and exposed for 20 minutes on a phosphor imaging plate (Fujifilm, Tokyo, Japan). After exposure, the phosphor imaging plate was scanned with an image plate scanner (Typhoon 5, GE Healthcare, Chicago, Illinois, USA). The total gamma counts of [^68^Ga]Ga-RGD in each slice of the heart was afterwards measured using a gamma counter (1400 Wizard-3, Perkin Elmer, Waltham, Massachusetts, USA). ImageJ (Fiji, NIH, USA) was used to evaluate the autoradiography. A region of interest (ROI) was drawn around each point on the dilution series and each slice depictured on the phosphor plate. The intensity of the dilution series was converted into MBq and the maximum MBq of [^68^Ga]Ga-RGD in each slice of the heart were estimated. For analyses of gamma counting, the total counts of [^68^Ga]Ga-RGD were corrected for dose, injection time and weight of the slice (%ID/g).

*Histology*

After gamma counting, the slices were fixed in 4% formaldehyde and embedded in paraffin. Short axis slides of 4 µm thickness were stained for collagen deposition (Masson’s Trichrome (MT)) and myocardial differentiation (Hematoxylin & Eosin (HE)). Furthermore, immunohistochemically staining was performed per subunit of integrin CD61 (Integrin β_3,_ 1:200, Sigma-Aldrich, St. Louis, Missouri, USA) and CD51 (Integrin α_V_, 1 μg/ml Thermo Fisher Scientific, Waltham, Massachusetts, USA).

Each slice was scanned using an Axio scan Z1 (Zeiss, Köln, Germany) and examined with software Zen 2012 (blue edition) (Zeiss, Köln, Germany). The slices stained with MT were quantified using a macro in ImageJ (Fiji, NIH, USA). The fibrotic areas were calculated as percentages of the whole heart areas.

*In vivo experiments:*

*Animal studies*

Experimental animals:

Outbred male Sprague-Dawley rats were used for the animal study (Janvier, Le Genest-Saint-Isle, France). The rats were six weeks old on arrival at our facility. The rats were acclimatized for 7-12 days before enrolment in the study. All animals were cared for at an on-site core animal facility. The animals had access to water and standard rodent chow ad libitum.

*Coronary occlusion:*

The rats were initially anaesthetized with 4% Sevoflurane and then intubated and ventilated (UNO micorventilator-O3, Zevenaar, Netherlands) with Sevoflurane 2.5% to 4% to ensure sedation. The rats were placed on a heated operation table during surgery. Prior to incision, the rats were treated with buprenorphine 0.05 mg/kg subcutaneously. The rats were postoperatively treated with buprenorphine 0.05 mg/kg four times/day in the first 24 hours and three times/day during the following 24-72 hours.

The chest of the animals was shaved, and iodine used for sterilization. After ensuring sterile surgical conditions, an incision was made in the skin and a thoracotomy was performed at the third or fourth intercostal space, depending on size of the rat. The pericardium was identified and opened. The LAD was identified and permanently ligated 5 mm distal to branching off the left coronary artery with a 5-0 polypropylene suture. The myocardium was observed for ischemia. If there was no discoloration of the myocardium, another suture of the LAD was made until discoloration of the myocardium confirmed ischemia. After confirmation, vicryl 4-0 suture was used to close the thoracotomy, muscle layer and skin. To eliminate a potential bias in the data due to wound healing of the thorax, sham animals also underwent thoracotomy, but without LAD ligation.

*Positron emission tomography imaging:*

Out of the 104 animals included, 16 animals did not survive the surgery (15 %) and did not undergo PET/CT. The remaining 88 animals underwent a PET/CT scan one week after surgery (N: MI=55, sham=33). Follow-up scans was performed four weeks after surgery (N: MI=23, sham=13). PET/CT imaging was performed using a preclinical PET/CT scanner (Siemens Inveon, Knoxville, TN, USA).

*Tracer preparation:*

The tracers were produced at the PET & Cyclotron Unit at Copenhagen University Hospital (Rigshosptalet, Copenhagen, Denmark).

[^68^Ga]Ga-RGD tracer preparation:

Labelling of NODAGA-RGD (ABX GmbH, Radeberg, Germany) dimer was performed by mixing 60 µg peptide with 1 ml of 0.7 M NaOAC, 5 mg/mL ascorbic acid, pH 5.0 followed by elution of ^68^Ge/^68^Ga generator (IGG100; Eckert & Ziegler, Berlin, Germany) with 4 mL of 0.1 M HCl. The reaction was performed at 60°C for 5 min with at RCP ≥ 91 %.

For quality analysis, a high-performance liquid chromatograph (Ultimate 3000; Dionex, Thermo fischer, Waltham, Massachusetts, USA)) was performed on a kinetex C18 column (2.6μm, 100-Å, 50 × 4.6 mm; Phenomenex, Værløse, Denmark) and with the UV and radiodetector connected in series. The mobile phases were eluent A, 0.1% trifluoroacetic acid in H2O, and eluent B, 0.1% trifluoroacetic acid in MeCN.

2-[^18^F]FDG tracer preparation:

The 2-[^18^F]FDG radiotracer was produced as the standard formulation for use in clinical diagnostics.

*[^68^Ga]Ga-RGD PET/CT:*

The rats were anaesthetized with 4% sevoflurane. A 24G intravenous catheter (Vasofix^®^ Safety, Braun, Denmark) was placed in the tail vein. Then, 0.6 mL of [^68^Ga]Ga-RGD tracer was administered, to ensure constant level [^68^Ga]Ga-RGD peptide (mean [^68^Ga]Ga-RGD dose 36 MBq ±19 MBq). During the PET/CT scan, the rats were monitored with ECG and respiration frequency. First, the rats underwent CT scan for attenuation correction. Prior to the CT scan, the rats were injected with 2 mL of CT contrast (Iomeron 350 mg I/mL, Bracco Imaging, Gothenburg, Sweden) to identify the myocardial wall. Thirty minutes after [^68^Ga]Ga-RGD tracer injection, the rats underwent a minimum of fifteen minutes PET acquisition. The duration of the PET acquisitions was prolonged if the rats had been injected with less than 40 MBq of [^68^Ga]Ga-RGD. This was done to ensure a sufficient number of true counts. The images were reconstructed using ordered Poison – maximum a posteriori probability (OP-MAP)/3D-ordered subset expectation maximization (OSEM) algorithm with a 2 mm requested resolution, 2 iteration and 18 subsets. The images were scatter, attenuation and prompt-gamma corrected.

Inveon Research Workplace (version 4.2, Siemens, Knoxville, TN, USA) was used to analyze PET data. The CT was used to draw three circular ROIs: one in the infarcted area – corresponding to the anterior wall of the myocardium, one in the healthy area – corresponding to the posterior wall of the myocardium, and one in the left ventricle blood pool. These ROIs were then copied onto the spatially aligned PET images. It is visually checked that the uptake in the ROIs are not influenced by spill-over from adjacent tissue (Figure S4). The quantitative [^68^Ga]Ga-RGD uptake from each ROI was calculated as mean and maximal percentage injected dose per gram (%ID/g). The target-to-background ratio was calculated as the injected dose per gram in the anterior wall compared to the injected dose per gram in the posterior wall.

*2-[^18^F]FDG PET/CT:*

Six hours before imaging, access to food was restricted in order to ensure metabolic conditioning. Fifteen minutes before 2-[^18^F]FDG-injection, 15 mg/kg acipimox (Sigma-Aldrich, St. Louis, Missouri, USA) were subcutaneously administered which reduces free fatty acid levels facilitating cardiac FDG-uptake. A 24G intravenous catheter was placed in the tail vein with a 24G catheter (Vasofix^®^ Safety, Braun, Denmark) and 60-70 mBq 2-[^18^F]FDG was injected. During the PET/CT scan, the rats were monitored with ECG and respiration frequency. The rats underwent a CT-scan for attenuation correction, and 60 minutes after the 2-[^18^F]FDG injection, the rats underwent a 15-minute PET scan. The static images were reconstructed using ordered Poison – maximum a posteriori probability (OP-MAP)/3D-ordered subset expectation maximization (OSEM) algorithm with a 1,5 mm requested resolution, 2 iteration and 18 subsets. The images were scatter, attenuation and prompt-gamma corrected. The gated images were reconstructed as an 8 bin gated image using the same reconstruction algorithm as described above.

The software package Corridor4DM version 2017 (Invia LLC, Ann Arbor, Michigan, USA) was used to analyze the cardiac 2-[^18^F]FDG –PET images. The PET images were automatically reoriented into short, vertical and horizontal long axes and the myocardial contour were outlined automatically. Both reorientation and contouring were visually inspected and corrected by two experienced certified consultants in nuclear medicine in conjunction. If the images were of poor quality, the investigators would exclude the PET/CT.

The 2-[^18^F]FDG -PET was analyzed using the American Heart Association (AHA) 17 segments model and metabolic defects calculated for every segment, resulting in a summed score. The gated images were used to analyze end diastolic volume (EDV), end systolic volume (ESV) and ejection fraction (EF),

*Statistical analysis:*

Data are presented as mean ± standard error of the mean (SEM), if not stated otherwise. All statistical analyses were performed using SPSS version 26 (IBM SPSS, Chicago, IL, USA). For comparison, the Student t-test was used. For association between variables, Fisher’s exact test was used. For correlation analysis, Spearman’s rank test was used. A multiple regression analysis was performed to investigate if end diastolic volume 4 weeks after MI could be predicted by the predictors; [^68^Ga]Ga-RGD uptake (%ID/g), LVEF and FDG extent 1 week after MI. A two-sided p-value < 0.05 was considered significant.

Reproducibility of %ID/g measurement was assessed using the intraclass correlation coefficient (two-way mixed effects model with absolute agreement) with corresponding 95%-confidence intervals (95% CI). All scans were used for reproducibility measurements.

**Table S1: SPR kinetics analysis of integrin α_v_β_3_ the three analytes**

| Integrin α_v_β_3_ | Divalent cation | Kinetic fit | k_on_ (M^-1^s^-1^) | k_off_ (s^-1^) | KD ()M | χ^2^ |
| --- | --- | --- | --- | --- | --- | --- |
| [68Ga]Ga-RGD | Mg^2+^ | Kinetic | 9,3 x 10^+5^ | 7,0 x 10^-5^ | 7,5 x 10^-11^ | 0,10 |
| Vitronectin | Mg^2+^ | Kinetic | 3,2 x 10^+4^ | 2,1 x 10^-4^ | 1,3 x 10^-9^ | 0,49 |
| Fibronectin | Mg^2+^ | Steady state |  |  | 2,1 x 10^-8^ | 0,20 |
|  |  |  |  |  |  |  |
| [68Ga]Ga-RGD | Mg^2+^ + Mn^2+^ | Kinetic | 3,5 x 10^+5^ | 2,5 x 10^-7^ | 7,1 x 10^-13^ | 0,20 |
| Vitronectin | Mg^2+^ + Mn^2+^ | Kinetic | 1,3 x 10^+4^ | 3,2 x 10^-4^ | 2,4 x 10^-8^ | 1,30 |
| Fibronectin | Mg^2+^ + Mn^2+^ | Steady state |  |  | 7,2 x 10^-8^ | 0,47 |

| Panel |  |
| --- | --- |
| BV421 CD61, integrin β_3_ chain | BD Biosciences, Kongens Lyngby, Denmark |
| PE CD51, integrin α_v_ chain | Biolegend, San Diego, California, USA |
| BB515 CD31 | BD Biosciences, Kongens Lyngby, Denmark |
| BV480 CD45 | BD Biosciences, Kongens Lyngby, Denmark |
| BV711 CD11b/c | BD Biosciences, Kongens Lyngby, Denmark |
| BV786, anti granulocytes, clone RP-1 | BD Biosciences, Kongens Lyngby, Denmark |
| APC MHC-II | BD Biosciences, Kongens Lyngby, Denmark |
| eFLOUR 780 and viability dye | Thermo Fischer Scientific, Waltham, Massachusetts, USA |

**Table S2: the 8-color flow cytometry-based optimized multicolor immunofluorescence panel used in flow cytometry**

**Figure legend**

**Figure S1**: Sensorgram from SPR and the fitted curves between integrin α_v_β_3_ and [^68^Ga]Ga-RGD, vitronectin and fibronectin.

**Figure S2**: Pearsons correlation matrix of parameters from flow cytometry and %ID/g mean [^68^Ga]Ga-RGD from PET/CT

**Figure S3**: Gating of flow cytometry data. All populations were gated on time, as singlets, as cells (based on scatter properties) and as viable. Endothelial cells were further defined as CD45- CD31+. Immune cells were further defined as CD45+ CD31-. Myocytes were further defined as CD45- CD31-.

Figure S4: The CT-scan (left) was used to draw ROIs in the anterior (blue), posterior (red) and blood volume (green). The ROIs was copied to the spatially aligned PET images (right).

**Figure S1:**


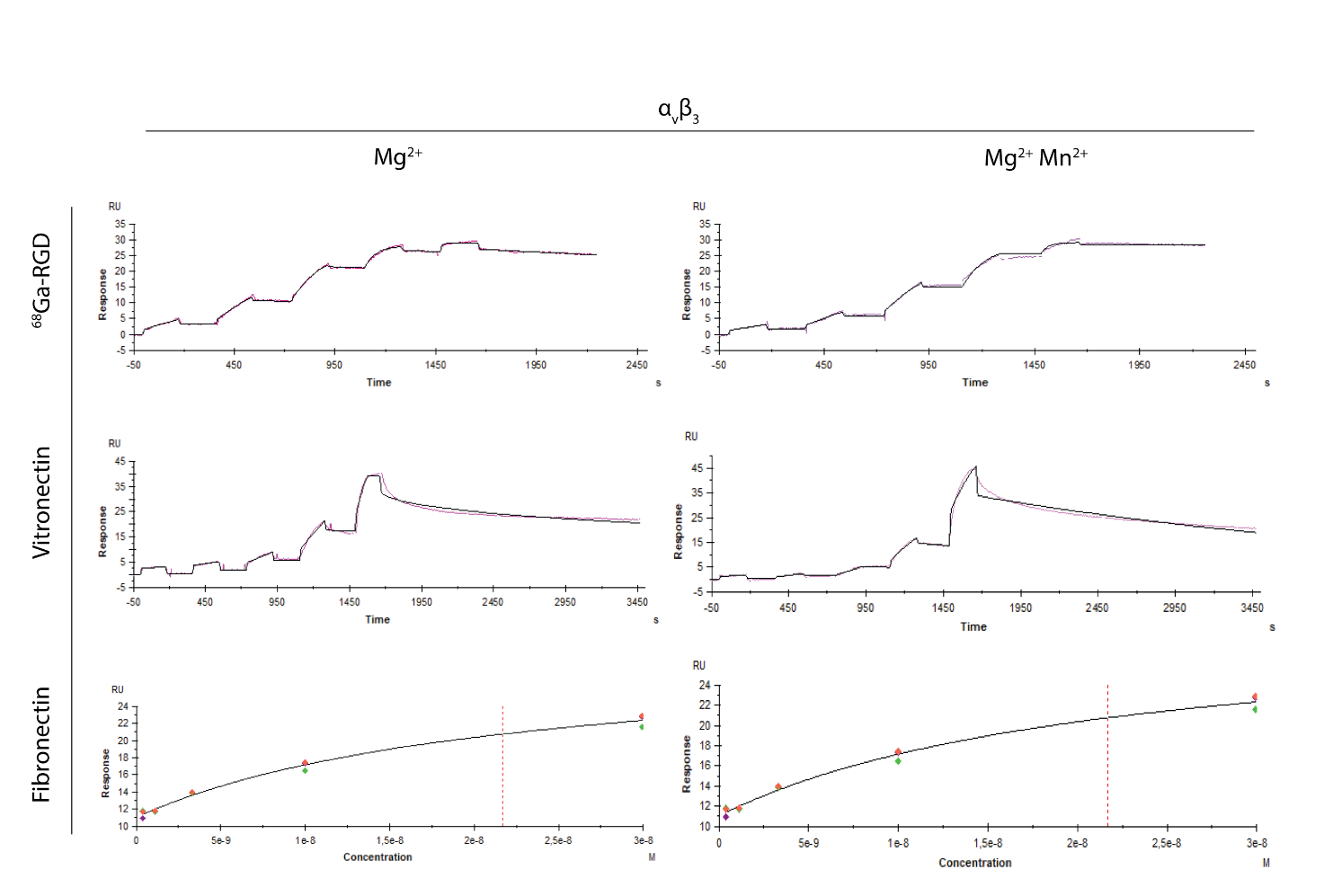


**Figure S2:**


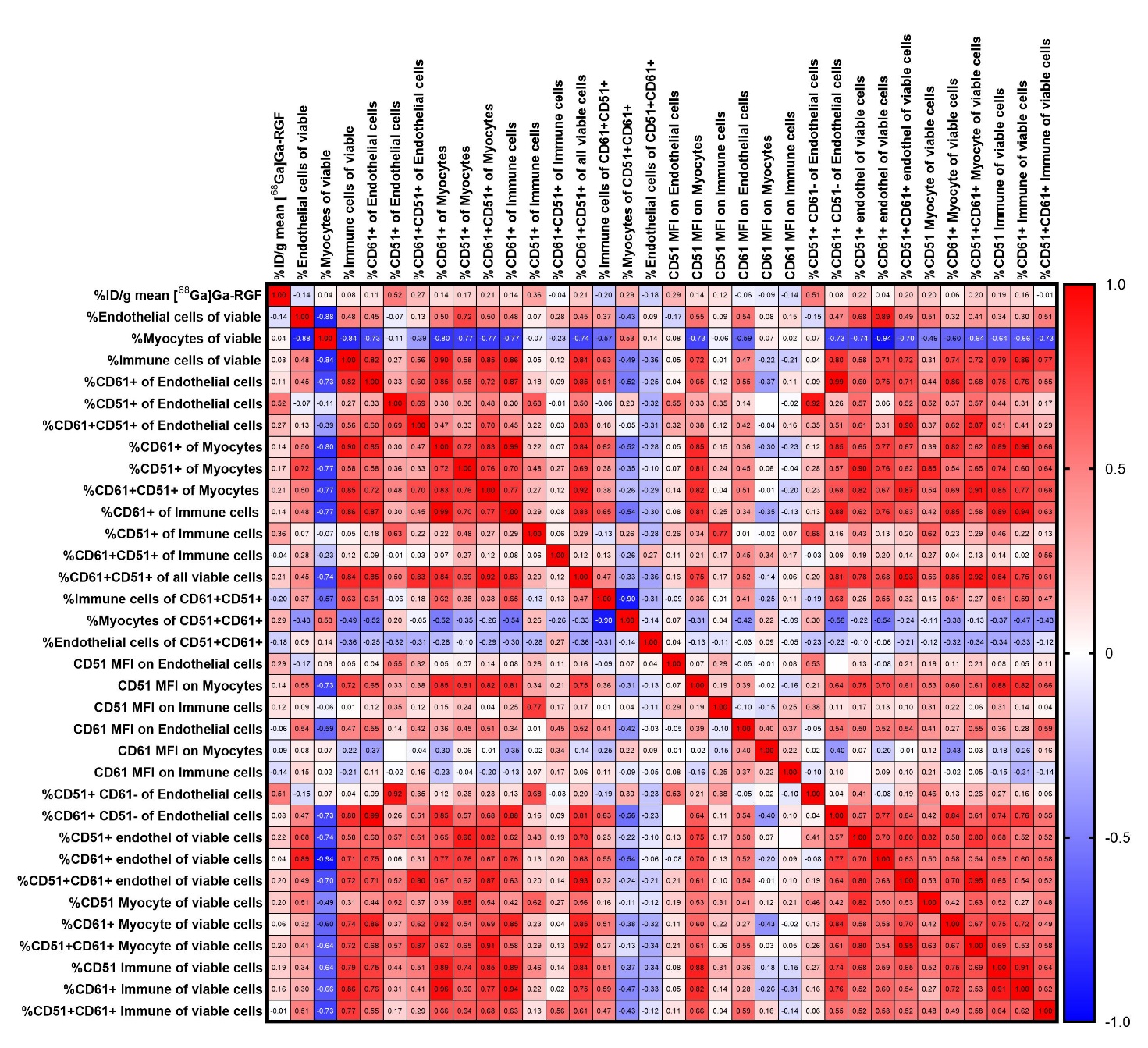


**Figure S3:**


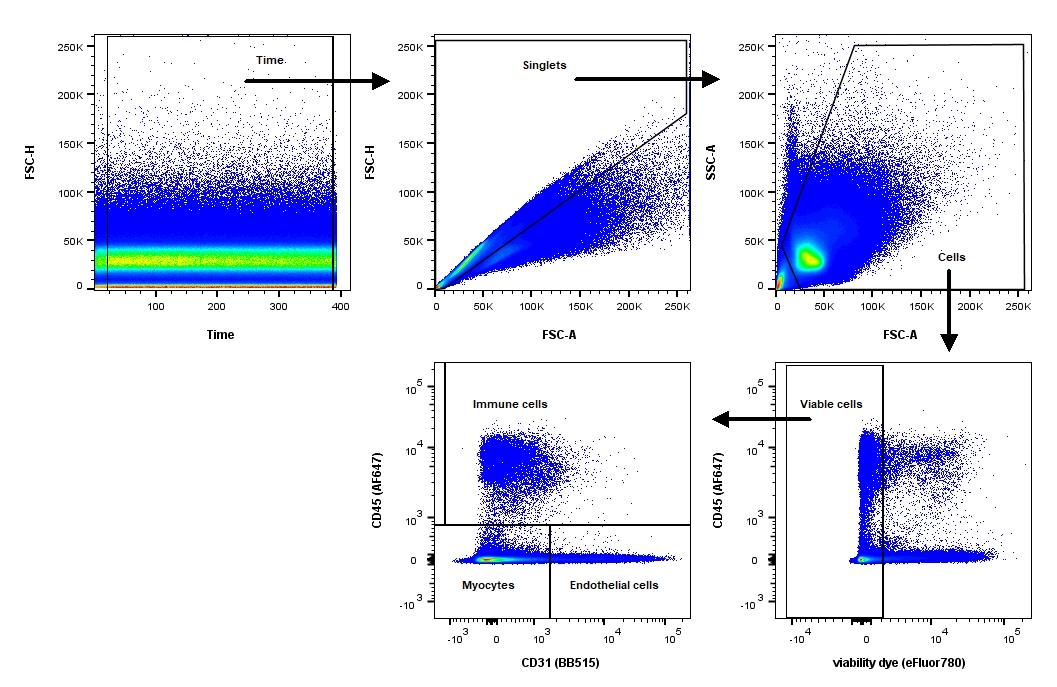


Figure S4


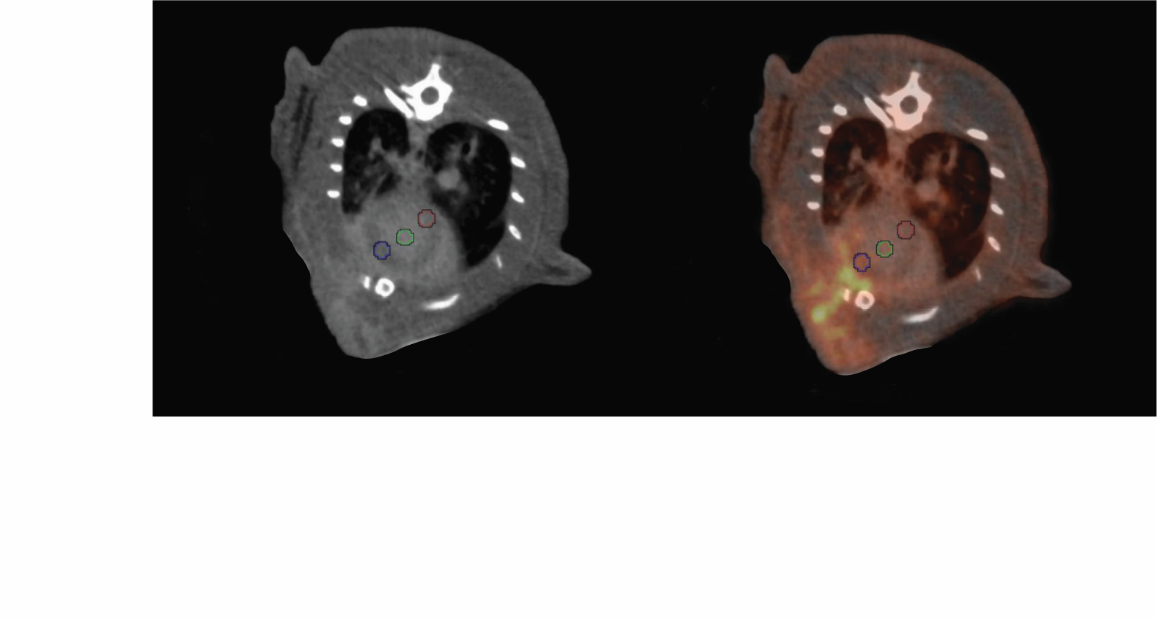

Supplement: Supplementary file 1 — Supplementary file1 (DOCX 1836 kb) [file 12350_2023_3265_MOESM1_ESM.docx]
