# Supplementary material for: [68Ga]Ga-NODAGA-E[(cRGDyK)]2 angiogenesis PET following myocardial infarction in an experimental rat model predicts cardiac functional parameters and development of heart failure
Source: J Nucl Cardiol. 2023 May 1;30(5):2073–84. doi: 10.1007/s12350-023-03265-9 (PMC10558373; doi:10.1007/s12350-023-03265-9)
Supplement: Supplementary file 2 — Supplementary file2 (PPTX 7627 kb) [file 12350_2023_3265_MOESM2_ESM.pptx]

## Slide 1
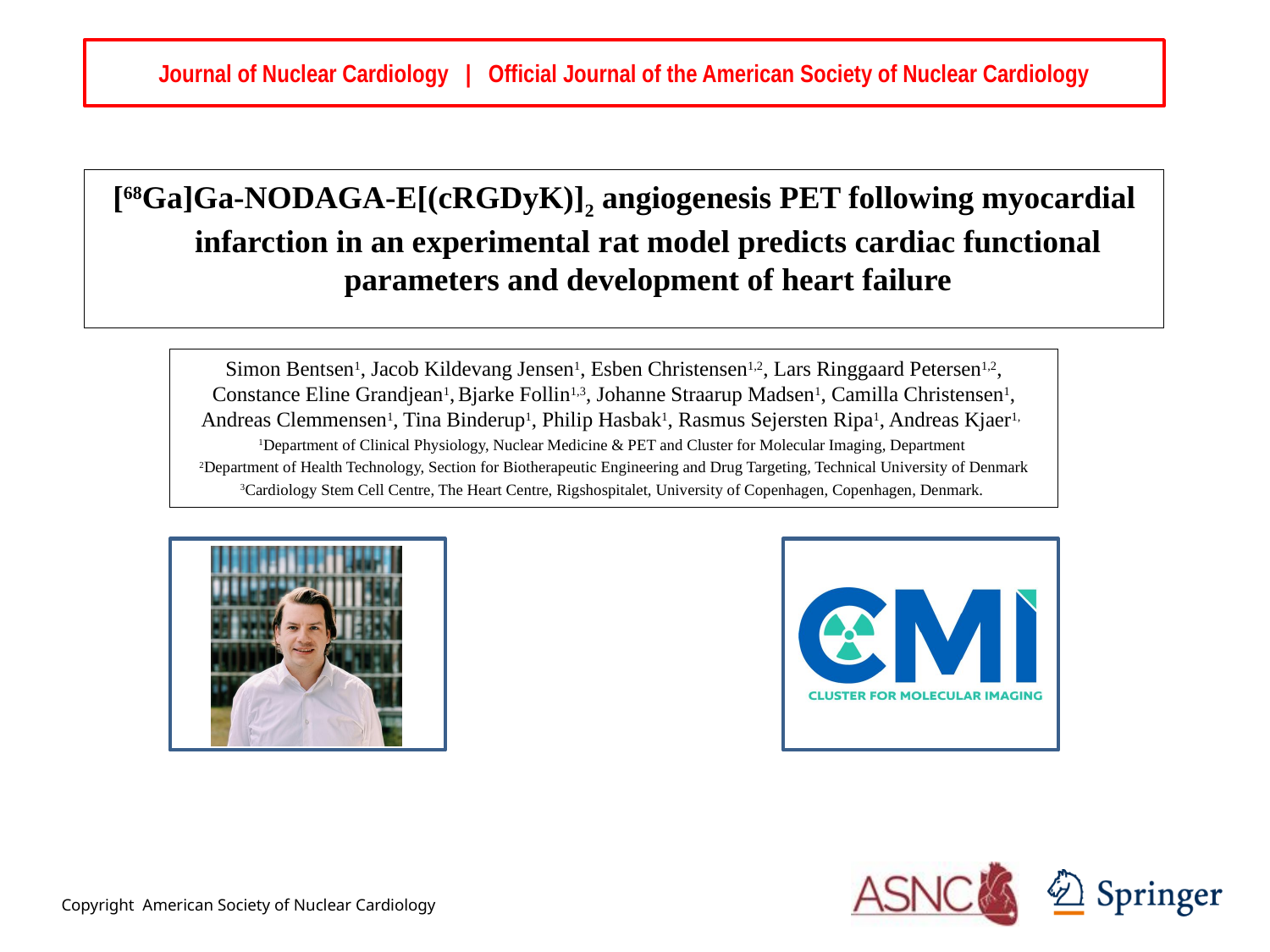

Journal of Nuclear Cardiology | Official Journal of the American Society of Nuclear Cardiology
# [68Ga]Ga-NODAGA-E[(cRGDyK)]2 angiogenesis PET following myocardial infarction in an experimental rat model predicts cardiac functional parameters and development of heart failure
Simon Bentsen1, Jacob Kildevang Jensen1, Esben Christensen1,2, Lars Ringgaard Petersen1,2, Constance Eline Grandjean1, Bjarke Follin1,3, Johanne Straarup Madsen1, Camilla Christensen1, Andreas Clemmensen1, Tina Binderup1, Philip Hasbak1, Rasmus Sejersten Ripa1, Andreas Kjaer1,
1Department of Clinical Physiology, Nuclear Medicine & PET and Cluster for Molecular Imaging, Department
2Department of Health Technology, Section for Biotherapeutic Engineering and Drug Targeting, Technical University of Denmark
3Cardiology Stem Cell Centre, The Heart Centre, Rigshospitalet, University of Copenhagen, Copenhagen, Denmark.
Institution
Picture/Logo
Optional
Copyright American Society of Nuclear Cardiology

## Slide 2
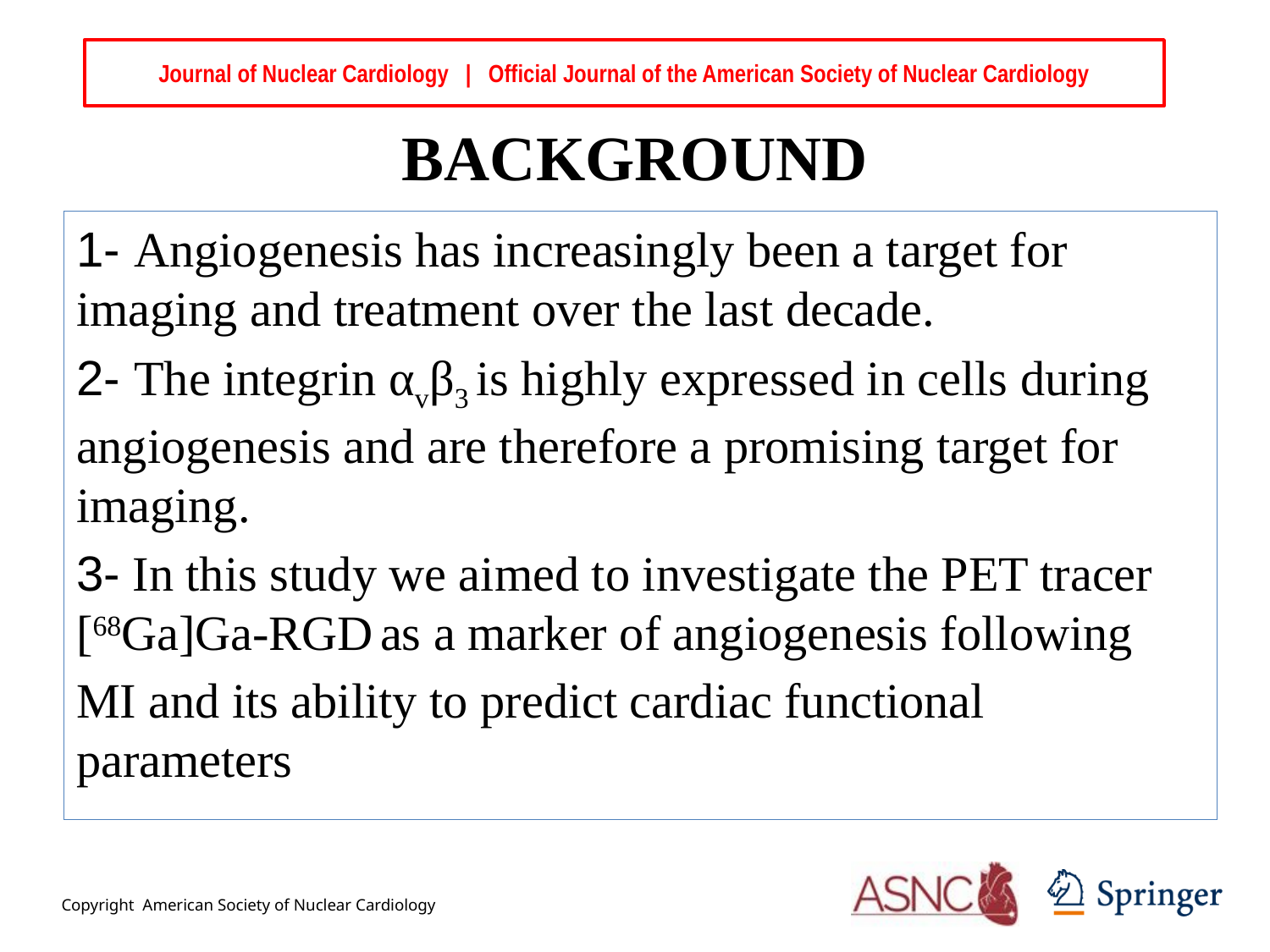

Journal of Nuclear Cardiology | Official Journal of the American Society of Nuclear Cardiology
# BACKGROUND
1- Angiogenesis has increasingly been a target for imaging and treatment over the last decade.
2- The integrin αvβ3 is highly expressed in cells during angiogenesis and are therefore a promising target for imaging.
3- In this study we aimed to investigate the PET tracer [68Ga]Ga-RGD as a marker of angiogenesis following MI and its ability to predict cardiac functional parameters
Copyright American Society of Nuclear Cardiology

## Slide 3
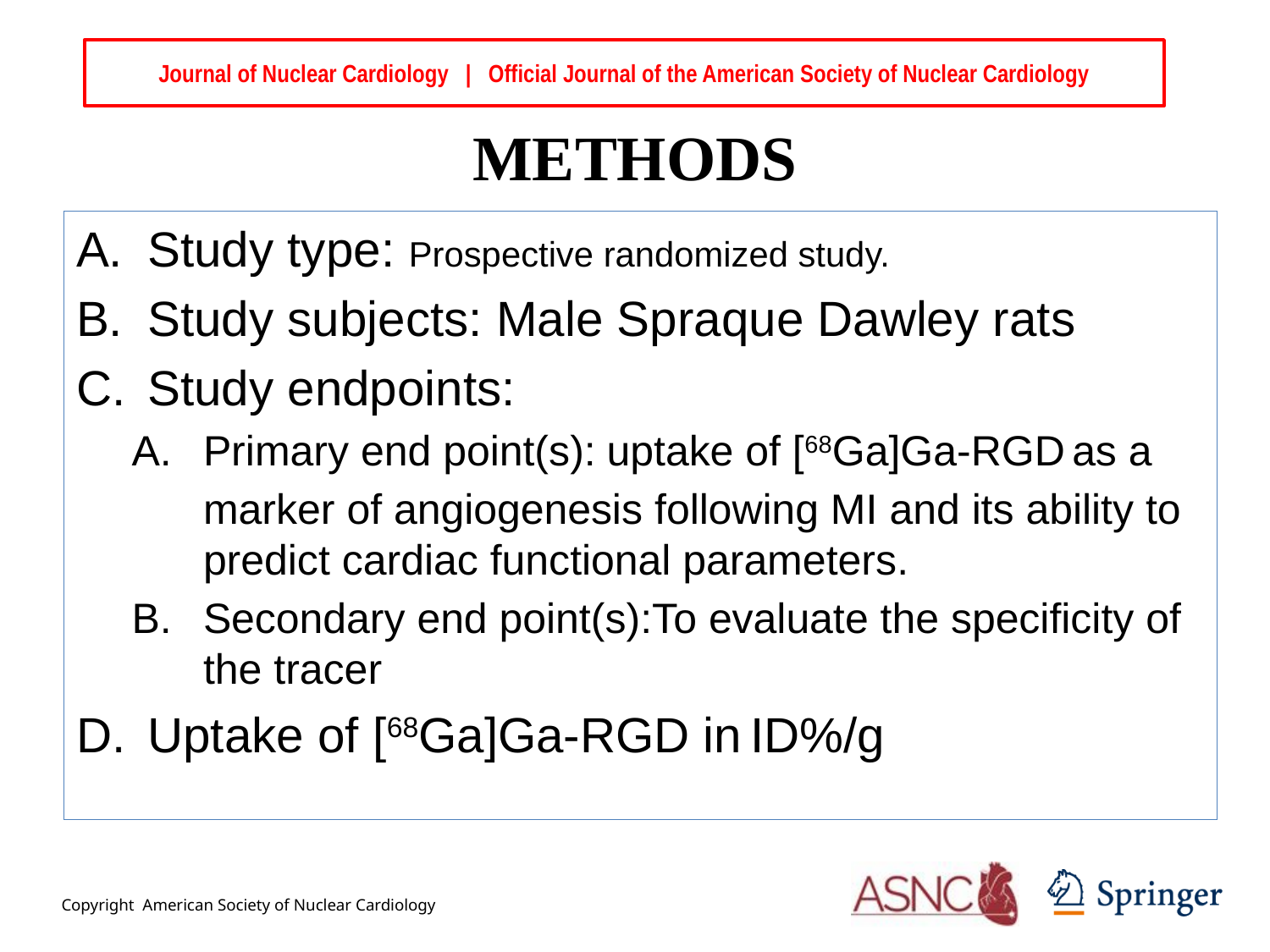

Journal of Nuclear Cardiology | Official Journal of the American Society of Nuclear Cardiology
# METHODS
Study type: Prospective randomized study.
Study subjects: Male Spraque Dawley rats
Study endpoints:
Primary end point(s): uptake of [68Ga]Ga-RGD as a marker of angiogenesis following MI and its ability to predict cardiac functional parameters.
Secondary end point(s):To evaluate the specificity of the tracer
Uptake of [68Ga]Ga-RGD in ID%/g
Copyright American Society of Nuclear Cardiology

## Slide 4
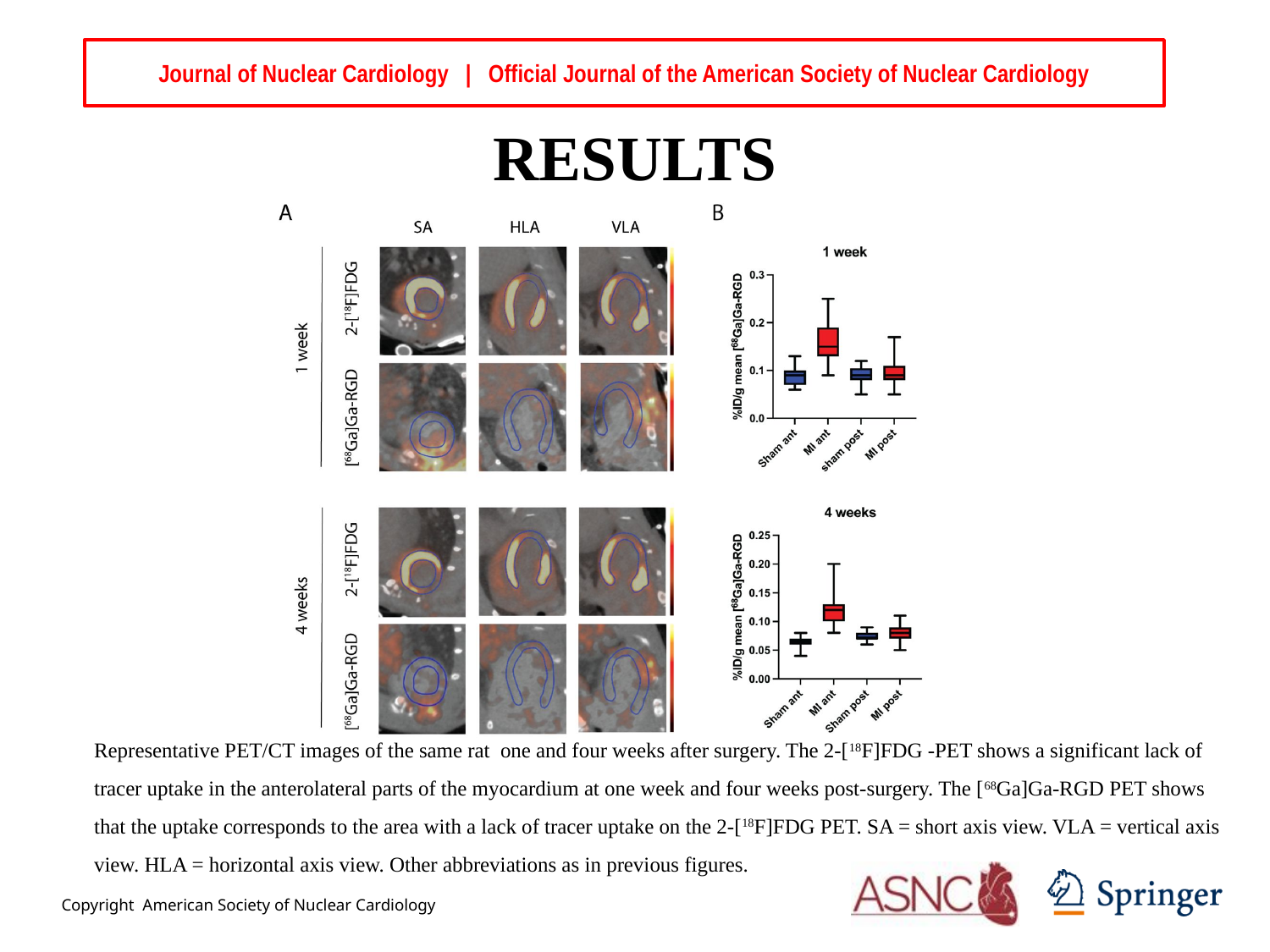

Journal of Nuclear Cardiology | Official Journal of the American Society of Nuclear Cardiology
# RESULTS
Representative PET/CT images of the same rat one and four weeks after surgery. The 2-[18F]FDG -PET shows a significant lack of tracer uptake in the anterolateral parts of the myocardium at one week and four weeks post-surgery. The [68Ga]Ga-RGD PET shows that the uptake corresponds to the area with a lack of tracer uptake on the 2-[18F]FDG PET. SA = short axis view. VLA = vertical axis view. HLA = horizontal axis view. Other abbreviations as in previous figures.
Copyright American Society of Nuclear Cardiology

## Slide 5
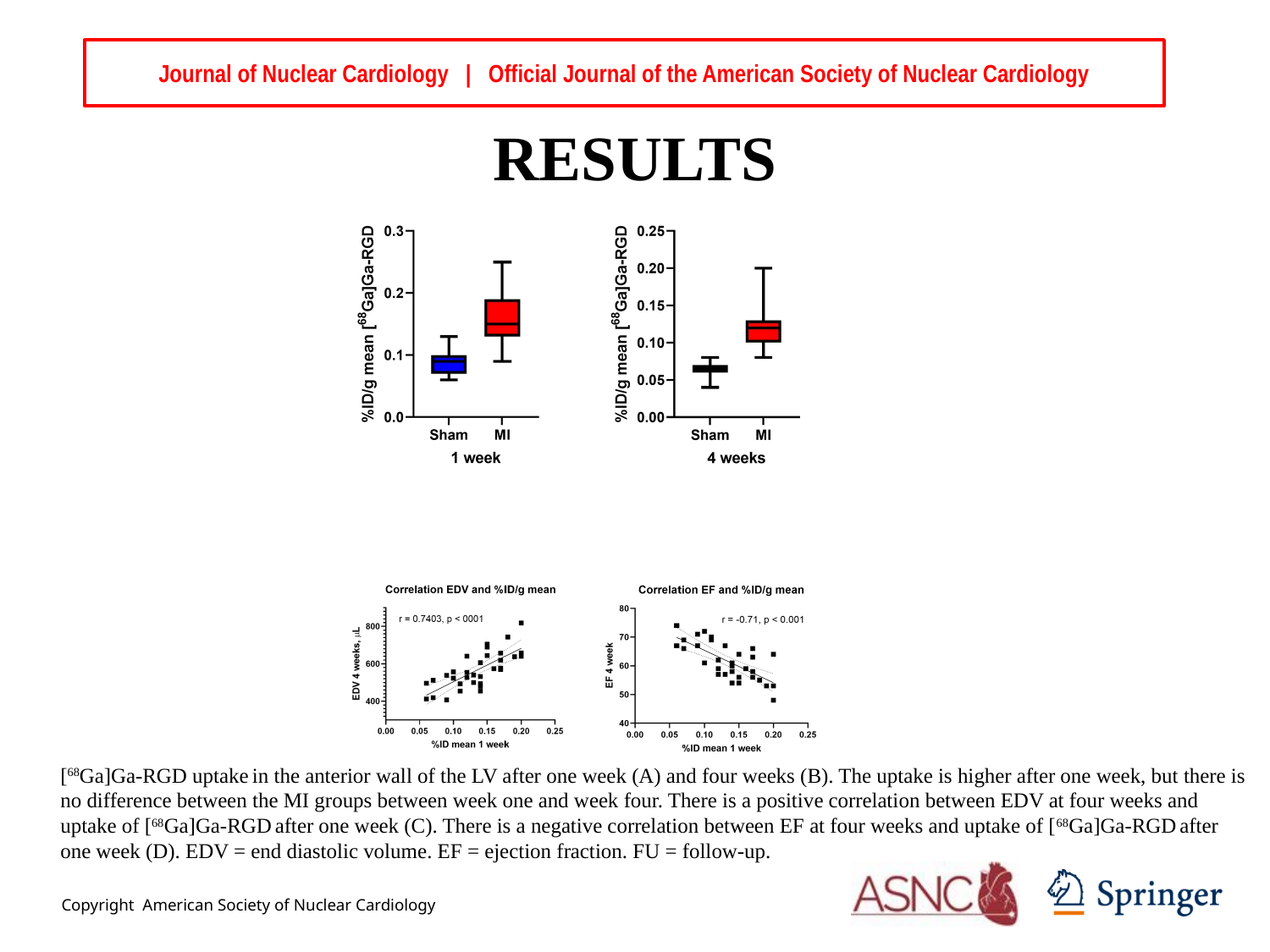

Journal of Nuclear Cardiology | Official Journal of the American Society of Nuclear Cardiology
# RESULTS
[68Ga]Ga-RGD uptake in the anterior wall of the LV after one week (A) and four weeks (B). The uptake is higher after one week, but there is no difference between the MI groups between week one and week four. There is a positive correlation between EDV at four weeks and uptake of [68Ga]Ga-RGD after one week (C). There is a negative correlation between EF at four weeks and uptake of [68Ga]Ga-RGD after one week (D). EDV = end diastolic volume. EF = ejection fraction. FU = follow-up.
Copyright American Society of Nuclear Cardiology

## Slide 6
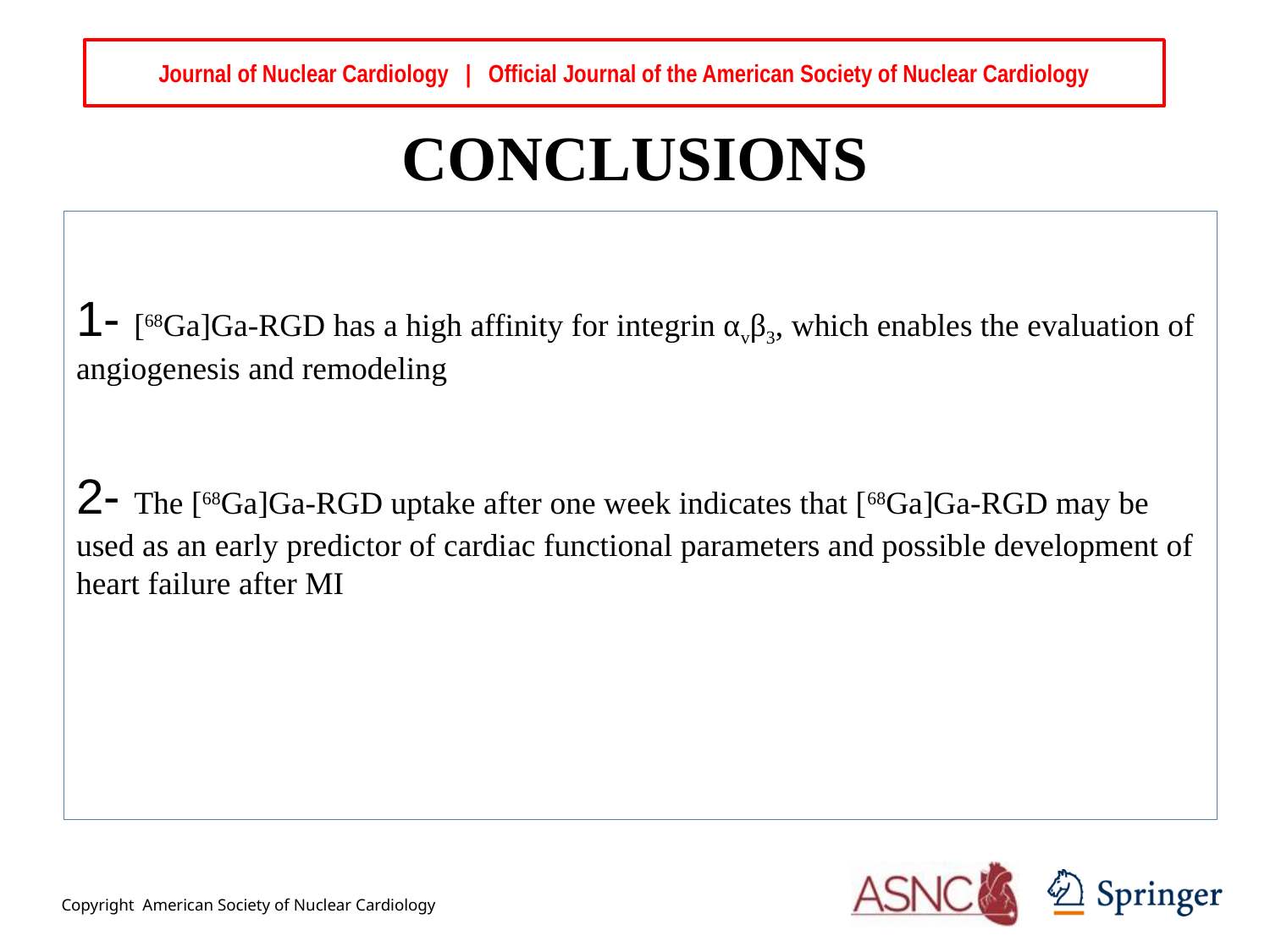

Journal of Nuclear Cardiology | Official Journal of the American Society of Nuclear Cardiology
# CONCLUSIONS
1- [68Ga]Ga-RGD has a high affinity for integrin αvβ3, which enables the evaluation of angiogenesis and remodeling
2- The [68Ga]Ga-RGD uptake after one week indicates that [68Ga]Ga-RGD may be used as an early predictor of cardiac functional parameters and possible development of heart failure after MI
Copyright American Society of Nuclear Cardiology
